# A highly alkaline-stable metal oxide@metal–organic framework composite for high-performance electrochemical energy storage

**DOI:** 10.1093/nsr/nwz137

**Published:** 2019-09-12

**Authors:** Shasha Zheng, Qing Li, Huaiguo Xue, Huan Pang, Qiang Xu

**Affiliations:** 1 School of Chemistry and Chemical Engineering, and Institute for Innovative Materials and Energy, Yangzhou University, Yangzhou 225009, China; 2 AIST-Kyoto University Chemical Energy Materials Open Innovation Laboratory, National Institute of Advanced Industrial Science and Technology, Kyoto 606-8501, Japan

**Keywords:** metal–organic framework, metal oxide, composite, electrochemical energy storage, flexible supercapacitor

## Abstract

Most metal–organic frameworks (MOFs) hardly maintain their physical and chemical properties after exposure to alkaline aqueous solutions, thus precluding their use as potential electrode materials for electrochemical energy storage devices. Here, we present the design and synthesis of a highly alkaline-stable metal oxide@MOF composite, Co_3_O_4_ nanocube@Co-MOF (Co_3_O_4_@Co-MOF), via a controllable and facile one-pot hydrothermal method under highly alkaline conditions. The obtained composite possesses exceptional alkaline stability, retaining its original structure in 3.0 M KOH for at least 15 days. Benefitting from the exceptional alkaline stability, unique structure, and larger surface area, the Co_3_O_4_@Co-MOF composite shows a specific capacitance as high as 1020 F g^−1^ at 0.5 A  g^−1^ and a high cycling stability with only 3.3% decay after 5000 cycles at 5 A g^−1^. The as-constructed solid-state flexible device exhibits a maximum energy density of 21.6 mWh cm^−3^.

## INTRODUCTION

Metal–organic frameworks (MOFs) are formed via self-assembly of metal ions and organic linkers [1–3]. Due to their superior properties, such as their large surface area, high porosity and structure tunability, MOFs have recently emerged as one type of important porous materials and have attracted intense interest in many fields, such as gas storage and separation [4–7], catalysis [8–11] and energy storage [12–15]. Nevertheless, MOFs still have a few weak points, which impede the use of their full potential to a great extent. For example, most MOFs manifest inferior properties for electrical conduction and have limited chemical stability (in water, especially alkaline conditions), preventing them from exhibiting their best performance in the field of electrochemistry [16–19]. Fortunately, hybridizing MOFs with a variety of functional materials to generate MOF composites can integrate the merits and mitigate the shortcomings of both parent materials [20–23].

Metal oxide nanomaterials with controllable shape, size, crystallinity and functionality are widely applied in many fields [24–27]. Because of their high theoretical specific capacitance, low cost and great reversibility, they are considered ideal pseudocapacitive electrode materials, but they have high surface energies and are prone to aggregation, leading to loss of the pseudocapacitive performance [28–30]. In addition, metal oxides usually display only small surface areas, which has largely restricted their use as electrode materials for electrochemical energy storage [31,32]. Consequently, finding a cost-effective method to increase the specific surface areas of metal oxides is crucial for achieving high pseudocapacitive activity.

Here, we report a strategy to integrate the advantages of both metal oxides and MOFs by hybridizing metal oxides with MOFs having large surface areas, in which each component retains its own identity while contributing extraordinary characteristics to the whole system [33,34]. MOFs with high surface areas provide appropriate spaces for the electrochemical reaction and intercalation/de-intercalation of cations (e.g. Li^+^, Na^+^, K^+^ and H^+^) during energy storage processes [13,35,36], while the presence of metal oxides effectively increases redox active sites [37–39]. We have successfully synthesized Co-MOF sheet (Co-MOF, Co_2_(ptcda)·2H_2_O, ptcda = perylene-3,4,9,10-tetracarboxylic dianhydride) through a simple one-pot hydrothermal method from the coordination of ptcda and Co^2+^. Interestingly, Co_3_O_4_ nanocubes grow on the surface of the Co-MOF sheet, forming the Co_3_O_4_@Co-MOF composite at pH = 11–13, which shows exceptional alkaline stability in 3.0 M KOH. Using the Co_3_O_4_@Co-MOF composite as the electrode for a supercapacitor, the specific capacitance reaches 1020 F g^−1^ at 0.5  A  g^−1^ in 3.0 M KOH. It shows a high rate capability with more than 96.7% capacitance retention even at 5 A g^−1^. In addition, an aqueous/solid-state flexible electrochemical capacitor energy storage device has been successfully fabricated using Co_3_O_4_@Co-MOF and activated carbon (AC), which displays high capacity and excellent cycling stability.

**Figure 1.**
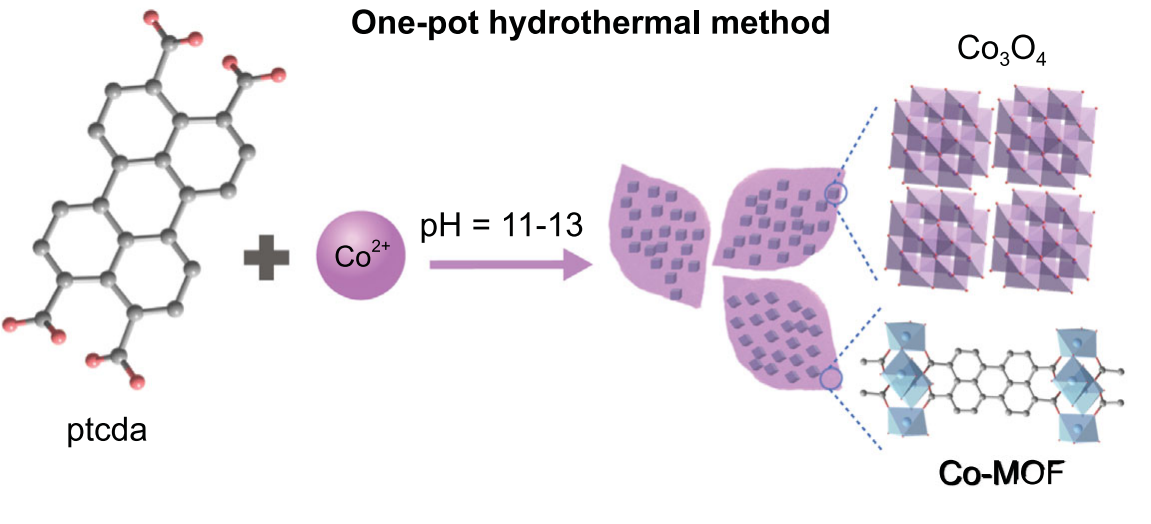
Schematic illustration of one-pot hydrothermal synthesis of Co_3_O_4_@Co-MOF composite.

## RESULTS AND DISCUSSION

**Figure 2.**
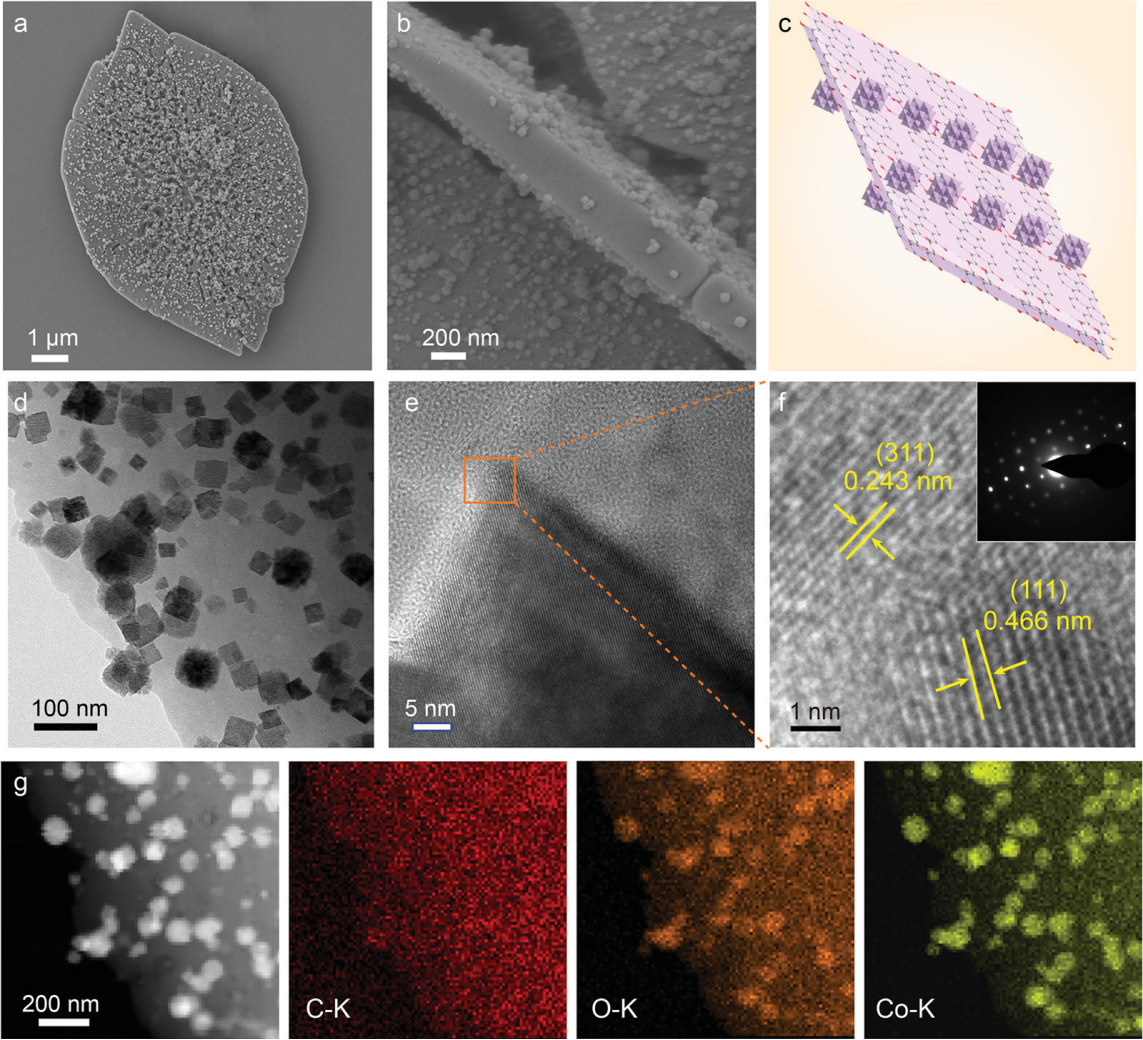
Microscopic characterization. (a, b) SEM images, (c) schematic morphology and (d) TEM image of Co_3_O_4_@Co-MOF. (e, f) High-resolution transmission electron microscope (HRTEM) images of Co_3_O_4_ (inset of (f): SAED pattern). (g) HAADF-STEM image of Co_3_O_4_@Co-MOF and the corresponding elemental mappings of C–K, O–K and Co–K.

The Co_3_O_4_@Co-MOF composite was synthesized through a one-pot solvothermal method (Fig. 1). The reaction of ptcda (C_24_H_8_O_6_) and cobalt acetate tetrahydrate (Co(CH_3_COO)_2_·4H_2_O) (C_24_H_8_O_6_:Co(CH_3_COO)_2_·4H_2_O = 1:1) in water at 100°C for 12 h with a C_24_H_8_O_6_:NaOH ratio of 1:4 affords leaf-like Co-MOF sheet (Co-MOF, Co_2_C_24_H_8_O_6_(OH)_4_, ∼5 μm in width and 8 μm in length), which has been confirmed by scanning electron microscopy (SEM) measurements (see Fig. S1c, d). Keeping the amount of C_24_H_8_O_6_ and Co(CH_3_COO)_2_·4H_2_O unchanged, a decrease in the C_24_H_8_O_6_:NaOH ratio to 1:2 gives a mixture of Co-MOF and unreated ptcda (Co-MOF + ptcda), while an increase in the C_24_H_8_O_6_:NaOH ratio to 1:6 results in the formation of a composite of Co_3_O_4_ nanocubes and Co-MOF (Co_3_O_4_@Co-MOF) (Fig. 2a; Fig. S1a, b, e, f). Under the same reation conditions, the reation of Co(CH_3_COO)_2_· 4H_2_O and NaOH with a molar ratio of 1:6 without ptcda produces Co_3_O_4_ nanocubes (see Fig. S2).

SEM and transmission electron microscopy (TEM) measurements of Co_3_O_4_@Co-MOF indicate that the Co_3_O_4_ nanocubes with an approximate average size of 50 nm are uniformly dispersed on both sides of Co-MOF (Fig. 2b–d). Fringes with lattice spacings of 0.243 and 0.466 nm for the (311) and (111) faces, respectively, along with the selected area electron diffraction (SAED) pattern, confirm the good crystalline characteristics of the Co_3_O_4_ nanocubes (Fig. 2e, f). The high-angle annular dark-field scanning TEM (HAADF-STEM) combined with elemental mapping measurements reveal that C, O and Co are distributed throughout the entire sheets (Fig. 2g). At the same time, the concentrations of O and Co dispersed on the nanocubes are relatively high. From these results, it was concluded that hybrid Co_3_O_4_ nanocubes were successfully synthesized on the Co-MOF. The color changes from red (Co-MOF + ptcda), reddish brown (Co-MOF) to black (Co_3_O_4_@Co-MOF) (see Fig. S3).

X-ray diffraction (XRD) measurements further confirm that the as-prepared composite is composed of Co-MOF and Co_3_O_4_ (JCPDS No. 42–1467) (see Fig. S4). The major diffraction peaks at 6.2, 7.1, 15.3, 19.3 and 22.0°, which can be indexed to the (001), (002), (110), (201) and (202) facets, respectively, of the Co-MOF, agree with those reported for the isostructural Zn-MOF with the formula Zn_2_(ptcda)·2H_2_O in the literature [40]. The structure analysis of Co-MOF is displayed in Fig. S5. The [CoO_6_] octahedral local structure leads to the connection of perylene cores to each other, forming a 3D open framework with a wavy layered structure. The formation of controllable interlayer spacing through the interaction between ptcda and Co^2+^ is beneficial to ion migration between organic layers. X-ray photoelectron spectroscopy (XPS) was performed to determine the chemical states of the Co, O and C elements of the as-obtained Co_3_O_4_@Co-MOF (see Figs S6–S8). The Co 2p spectra indicate that there are two types of Co species; the two fitting peaks at 780.6 and 796.3 eV are ascribed to Co^2+^, while another two fitting peaks at 779.3 and 794.5 eV are attributed to Co^3+^. The peaks at 780.6 and 796.3 eV are ascribed to the sum of Co^2+^ of Co_3_O_4_ and Co-MOF [41–43]. The O 1s and C 1s spectra of the samples indirectly verified the formation of Co_3_O_4_@Co-MOF (see Figs S7, S8). In addition, the Co_3_O_4_@Co-MOF composites exhibit a high Brunauer–Emmett–Teller (BET) surface area of 453.5 m^2^ g^−1^, which is remarkably larger than those of other samples (BET surface areas for Co-MOF + ptcda and Co-MOF are 313.6 and 445.2 m^2^ g^−1^, respectively) (see Fig. S9). As shown in Fig. S10, the pore size distribution for the Barrett–Joyner–Halenda (BJH) adsorption branch implies that the mesopores of the samples were below 20 nm. Furthermore, the pore size distribution was calculated through the Saito–Foley (SF) method, finding that the micropores were centered at 0.5–1 nm. These results clearly indicate the coexistence of micropores and mesopores in the samples. Therefore, the samples have a high specific surface area for better electrolyte permeation to access more redox active sites.

**Figure 3.**
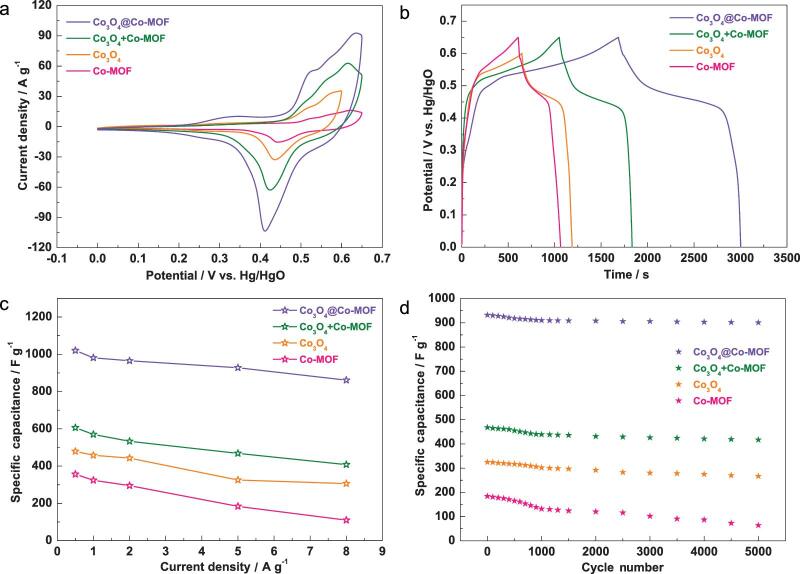
Electrochemical results of as-prepared electrodes (Co-MOF, Co_3_O_4_, Co_3_O_4_ + Co-MOF, Co_3_O_4_@Co-MOF) in a three-electrode cell in 3.0 M KOH aqueous solution. (a) CV curves with a scan rate at 30 mV s^−1^. (b) GCD curves at a current density of 0.5 A g^−1^. (c) The specific capacitance changing versus current densitiy from 0.5 A g^−1^ to 8 A g^−1^. (d) Cycling performance at 5 A g^−1^ for 5000 cycles.

The electrochemical capacitive properties of Co-MOF, Co_3_O_4_ and Co_3_O_4_@Co-MOF electrodes were evaluated in a three-electrode system in 3.0 M KOH. For comparison, we have also mechanically mixed Co_3_O_4_ and Co-MOF together with a mass ratio of 1:4 (the mass ratio calculation process is shown in Figs S11, S12 and Table S2), and the obtained mixture is called ‘Co_3_O_4_ + Co-MOF’ (see Fig. S13). From cyclic voltammetry (CV) curves of the as-obtained electrodes at different potentials and scan rates, the as-obtained electrodes mainly provide faradaic pseudocapacitive behavior, which is different from that of electric double-layer behavior (see Figs S14, S15) [44,45]. The surrounding area from the CV curve of the Co_3_O_4_@Co-MOF is larger than that of the Co-MOF, Co_3_O_4_ and Co_3_O_4_ + Co-MOF (Fig. 3a). These redox peaks come largely from the pseudocapacitance produced through faradaic redox reactions. However, the charge storage mechanism of the electrode material is still much less understood at the atomic level. The structural and valence changes of metal oxides/hydroxides during the charge/discharge process have recently been studied by *in situ* and *operando* observations, which offer novel insights into the energy storage mechanism of electrode material [46–48]. It is found that there is no large-scale structural evolution in the process of discharging and charging, but only a few minor migrations or adjustments of atom/ion species. Moreover, the highly reversible conversion of Co_3_O_4_/CoOOH can avoid morphological fractures caused by volume changes during cation de-intercalation/intercalation procedures. The possible reaction mechanism for Co-MOF and Co_3_O_4_ is as follows:


(1) \begin{eqnarray*} &&\rm{Co(II)}_{2}\rm{(ptcda)}\cdot 2\rm{H}_{2}\rm{O}+\rm{OH}^{-}\nonumber\\ &&\leftrightarrow\rm{Co(III)}_{2}{\rm {OH}(ptcda)}\cdot 2{\rm H}_{2}{\rm O} + {\rm e}^{-} \end{eqnarray*}

(2) \begin{equation*} {\rm {Co}}_{3}{\rm O}_{4}+{\rm OH}^{-}+{\rm H}_{2}\rm{O}\!\leftrightarrow\! 3\rm{CoOOH} + {\rm e}^{-} \end{equation*}

(3) \begin{equation*} \rm{CoOOH}+ {\rm OH}^{-}\leftrightarrow {\rm {CoO}}_{2}+{\rm H}_{2}{\rm O}+ {\rm e}^{-} \end{equation*}

**Figure 4.**
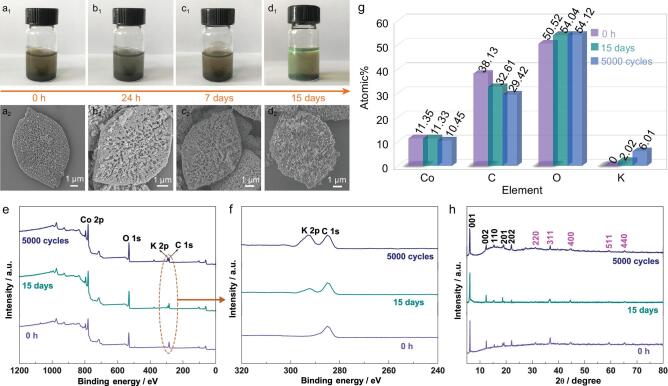
(a_1_-d_1_) Optical images of Co_3_O_4_@Co-MOF after immersion in 3.0 M KOH for 0 h, 24 h, 7 days and 15 days. (a_2_–d_2_) The corresponding SEM images. (e, f) XPS spectra and (g) contents of Co, C, O, K. (h) XRD patterns of Co_3_O_4_@Co-MOF for 0 h, 15 days and after cycling for 5000 cycles.

To evaluate the electrochemical properties of the as-obtained electrodes, galvanostatic charge–discharge (GCD) curves were generated (Fig. 3b). At 0.5 A g^−1^, the MOF-based materials possess high charge–discharge voltages (0.65 V), which are higher than that of Co_3_O_4_ (0.6 V). In addition, the Co_3_O_4_@Co-MOF displays the longest charge–discharge time. The specific capacitances were calculated from the GCD curves (see Fig. S16) of the as-obtained electrodes at 0.5, 1, 2, 5 and 8 A g^−1^ (Fig. 3c; the calculation is shown in the online supporting information). We can see that the specific capacitance of the Co_3_O_4_@Co-MOF composites (1020 F g^−1^) is much higher than those of Co_3_O_4_ + Co-MOF (606 F g^−1^), Co_3_O_4_ (479 F g^−1^) and Co-MOF (356 F g^−1^) at 0.5 A g^−1^, as well as those of the most recently reported metal oxides [49–51], MOFs [12,52,53] and MOF composites [54] (see Tables S3–6). Interestingly, the Co_3_O_4_@Co-MOF offers excellent rate capability through retaining a capacitance of 861 F g^−1^ when the current density increases 16 times (8 A g^−1^). Even at the current density of 32 A g^−1^, the capacitance can still reach 469 F g^−1^ (see Fig. S17), which proves the rate capability of Co_3_O_4_@Co-MOF to be as good as some recently reported high-performance MOF-based materials [46,52,55] (see Table S7). After 5000 cycles, the Co_3_O_4_@Co-MOF decayed only 3.1% compared with its initial capacity. Additionally, large decays were observed for Co_3_O_4_ + Co-MOF (10.9%), Co_3_O_4_ (17.8%), and Co-MOF (65.2%) (Fig. 3d). The conductivity of the as-obtained electrodes was also evaluated via electrochemical impedance spectroscopy (EIS) in the frequency range of 0.01–10^5^ Hz with open-circuit conditions (see Fig. S18). Moreover, the charge-transfer resistance (*R*_ct_) of the electrode was calculated by the Zsimp-Win software. The *R*_ct_ of Co_3_O_4_@Co-MOF was remarkably low, similar to those of Co_3_O_4_, Co-MOF and Co_3_O_4_ + Co-MOF. In addition, the curves showed that the *R*_ct_ after 5000 cycles is marginally larger than that of the original, which further evidences the stability of the composites.

To further investigate the chemical stabilities and understand the charge–discharge, SEM and TEM images of Co_3_O_4_@Co-MOF after cycling were obtained (see Figs S19–21). The morphology change of Co_3_O_4_@Co-MOF is negligible, and a number of nanopores can be found in the HRTEM image. Furthermore, the corresponding elemental mapping images of Co_3_O_4_@Co-MOF after cycling indicate that the element K was distributed in the entire Co-MOF, which may be due to the occurrence of K^+^ intercalation/de-intercalation in the MOF pores during charging/discharging. The chemical stabilities of Co-MOF and Co_3_O_4_@Co-MOF were checked in 3.0 M KOH. Co_3_O_4_@Co-MOF can retain the original morphology after immersion in 3.0 M KOH for 0 h, 24 h, 7 days and 15 days, whereas Co-MOF collapses even after only 24 h (Fig. 4a–d; Figs S22, S23). This is because of the preparation Co_3_O_4_@Co-MOF in strong alkaline conditions of (pH = 11–13) as well as the growth of highly stable Co_3_O_4_ on the surface of the Co-MOF, while Co-MOF was formed at pH = 6–8. In addition, Co-MOF is relatively more stable than Co_3_O_4_@Co-MOF in acidic solutions, which may be due to the different synthesis conditions (Figs S24, S25). Further, the XRD patterns and XPS spectra of Co_3_O_4_@Co-MOF after immersion in the alkaline solution for 0 h and 15 days and after cycling for 5000 cycles show that Co_3_O_4_@Co-MOFs can maintain their framework after cycling and immersion in the alkaline solution, which further confirms their good stability (Fig. 4e, f, h). After immersion and cycling, the element K was found to exist in Co_3_O_4_@Co-MOF, whose at% after cycling was three times that of composites after immersion (Fig. 4g; Fig. S26). Such an interesting phenomenon can be explained by the occurrence of K^+^ intercalation/de-intercalation in the MOF pores during charging/discharging and ion exchange in the solution, but for the immersion process, only ion exchange occurs.

**Figure 5.**
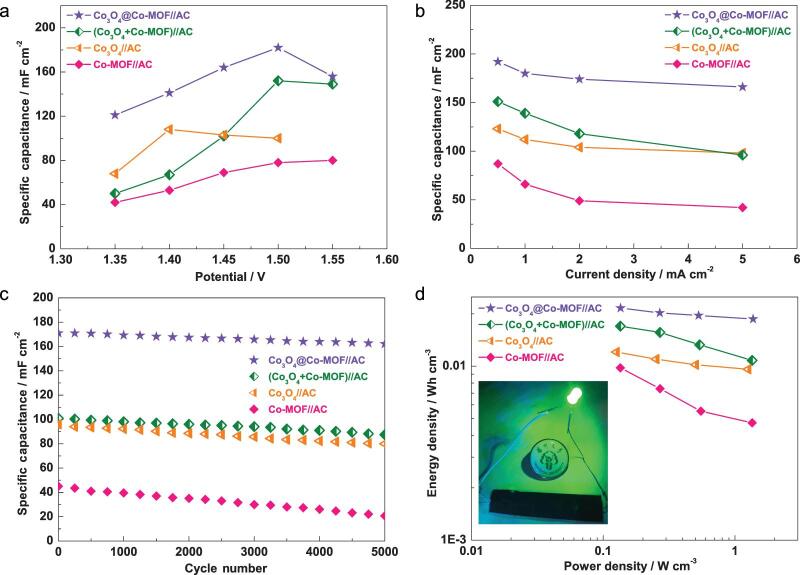
Electrochemical measurements of the as-prepared solid-state flexible devices. (a) Specific capacitance change versus potential. (b) Specific capacitance change versus current density. (c) Cycling property at 5 mA cm^−2^. (d) Ragone plot exhibiting the relationship between energy density and power density (inset of (d), optical image of the flexible device with solid-state electrolyte).

**Figure 6.**
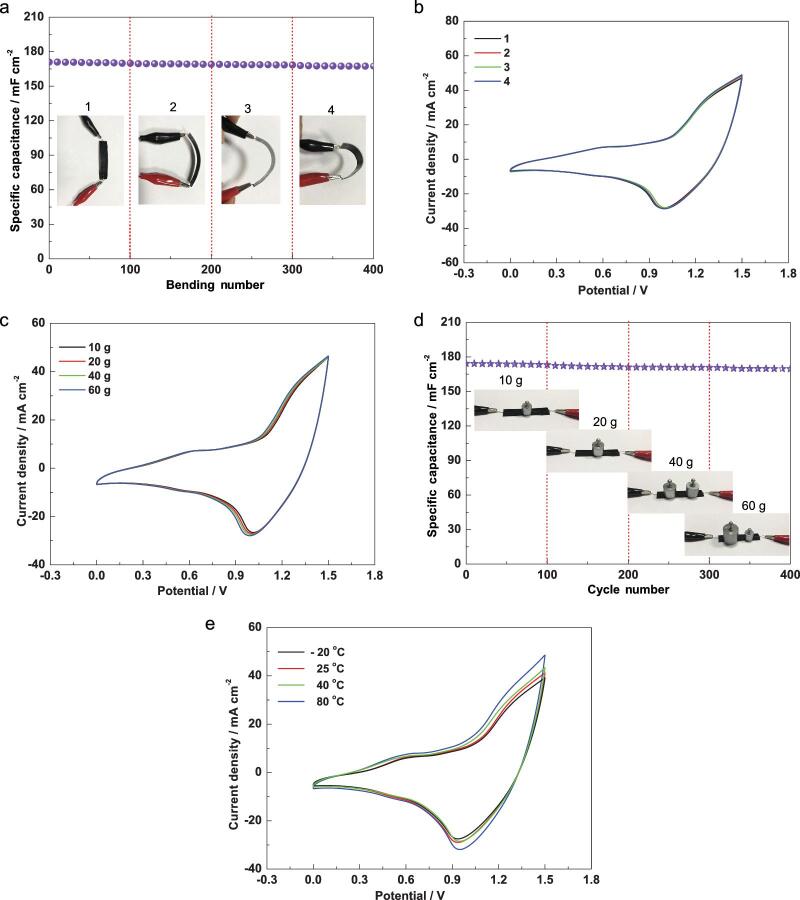
Electrochemical flexibility measurements of the as-prepared Co_3_O_4_@Co-MOF//AC solid-state flexible device. (a) Specific capacitance after 400 bending cycles with different bending degrees. (b) CV curves at 50 mV s^−1^ with four bending degrees. (c) CV curves under different load pressures. (d) Specific capacitance after 400 cycles under different load pressures. (e) CV curves at different temperatures.

Aqueous/solid-state flexible devices were constructed based on positive (the as-prepared nanomaterials) and negative (AC) materials according to the method that we have reported previously (supporting information) [32,54]. The specific capacitance of the activated carbon electrode was 168 F g^−1^ at 1.0 A g^−1^ (see Fig. S27). Based on the specific capacitance values and potential windows, the mass ratio between the positive and negative electrodes was set at 1:4 in the as-assembled device. In an aqueous electrolyte, the working potential range was expanded to 0–1.55 V (see Figs S28, S29). Figure S31a shows that there is more than one set of redox peaks in the CV curves, and the CV curves are not rectangular, perhaps because of Co_3_O_4_@Co-MOF for the surface redox mechanism of Co (II) to Co (III) from Co-MOF and Co_3_O_4_, respectively. The specific capacitance of the Co_3_O_4_@Co-MOF//AC aqueous device can reach 228 mF cm^−2^ at 0.5 mA cm^−2^, much higher than that of Co_3_O_4_ + Co-MOF//AC (163 mF cm^−2^), Co_3_O_4_//AC (129 mF cm^−2^) and Co-MOF//AC (97 mF cm^−2^) (see Figs S30, S31c). Based on the thickness of the electrode (see Fig. S32), the corresponding volumetric capacitances are obtained (Table S3). The specific capacitance of the Co_3_O_4_@Co-MOF//AC aqueous device is 96.2% of its initial capacitance after 5000 cycles (see Fig. S31d), which verifies the superb cycling property. Benefitting from the good conductivity of Co_3_O_4_, the Co_3_O_4_@Co-MOF//AC aqueous device has a lower *R*_ct_ (see Fig. S33), which agrees well with its good electrochemical property.

The solid-state flexible devices were also constructed via a facile method (see more details in the supporting information). All CV curves of the as-obtained solid-state flexible devices showed obvious oxidation and deoxidization peaks, indicating typical faradaic pseudocapacitive behavior (see Figs S34, S35). Specifically, the CV curves of the Co_3_O_4_@Co-MOF//AC solid-state flexible device retained their original shapes as the scan rate increased from 5 to 100 mV s^−1^, indicating their excellent rate performance. The specific capacitance change versus potential exhibits that the as-prepared MOF-based material solid-state flexible devices have the highest specific capacitance at 1.50 V, whereas, for the Co_3_O_4_//AC solid-state flexible device, the highest specific capacitance is located at 1.40 V (Fig. 5a; Fig. S36). The Co_3_O_4_@Co-MOF//AC solid-state flexible device exhibits a high specific capacitance of 192 mF cm^−2^ at 0.5 mA cm^−2^, which is remarkably higher than those of all other devices (specific capacitances for Co-MOF, Co_3_O_4_ and Co_3_O_4_ + Co-MOF are 87, 123 and 151 mF cm^−2^, respectively) (Fig. 5b; Fig. S37). Interestingly, at 5 mA cm^−2^, the Co_3_O_4_@Co-MOF//AC solid-state flexible device provides a superb rate capability via keeping the capacitance of 166 mF cm^−2^. After 5000 cycles, only 4.3% of the capacitance of the Co_3_O_4_@Co-MOF//AC solid-state flexible device was lost, which confirms the good cycling ability (Fig. 5c). Moreover, the Co_3_O_4_@Co-MOF//AC solid-state flexible device revealed a low *R*_ct_ value of 17 Ω, similar to that of Co_3_O_4_ (15 Ω). On the other hand, Co-MOF exhibited a slightly higher *R*_ct_ value of 23 Ω compared with Co_3_O_4_@Co-MOF, indicating that the combination of Co_3_O_4_ and Co-MOF effectively improved the electrical conductivity to some extent (see Fig. S38). The power density and energy density are crucial for actual application. The Co_3_O_4_@Co-MOF//AC solid-state flexible device indicated a peak energy density of 21.6 mW h cm^−3^ (Fig. 5d). Furthermore, the peak power density of the solid-state flexible device was 1373.2 mW cm^−3^ at 5 mA cm^−2^. The maximum energy density of the Co_3_O_4_@Co-MOF//AC solid-state flexible device was larger than those of all other devices. More importantly, the Co_3_O_4_@Co-MOF//AC solid-state flexible device was used to power a green light-emitting diode (LED). A green LED could be powered for approximately 4 min after charging for 30 s.

To measure the flexibility of the as-fabricated solid-state flexible device, the obtained solid-state flexible device was tested under different bending degrees for every 100 cycles. It is clear that the obtained solid-state flexible device lost only 0.28% under different bending degrees for 400 cycles (Fig. 6a), and TEM images of Co_3_O_4_@Co-MOF after 400 bending cycles were obtained (see Fig. S39). The morphology change of Co_3_O_4_@Co-MOF is negligible, which further confirms their excellent flexibility and stability. The CV curves under the four bending degrees are nearly unchanged, which further demonstrates that the obtained solid-state flexible device can work well under flexed conditions (Fig. 6b). Moreover, the environmental stability of the device was also examined by applying different pressures to the device. The CV curves with different load weights from 10 to 60 g change slightly (Fig. 6c). In the meantime, the obtained solid-state flexible device was tested under different load pressures for every 100 cycles, and the device demonstrated only 0.22% loss under different load pressures after 400 cycles (Fig. 6d). The device was measured at different temperatures from −20 to 80°C, but the area under the CV curve did not change much (Fig. 6e). Compared with that at room temperature (25°C), the area under the CV curve mildly increased at 80°C, while it very slightly decreased at −20°C. The reason for this might be the increased ion transport rate at elevated temperatures. When the temperature is higher than 80°C, the solid-state electrolyte changes into gel electrolyte, and thus we choose 80°C as the highest temperature.

## CONCLUSIONS

In summary, a composite of cobalt oxide nanocubes on Co-MOF sheet (Co_3_O_4_@Co-MOF) was successfully synthesized via a one-pot hydrothermal reaction under highly alkaline conditions. Without hybridizing with Co_3_O_4_, Co-MOF can provide an appropriate space for the electrochemical reaction and intercalation/de-intercalation of K^+^ during the energy storage process, but the alkaline stability of pristine Co-MOF is poor, resulting in capacitance as low as 356 F g^−1^. The presence of Co_3_O_4_ on the surface of Co-MOF effectively improves the alkaline stability, increases redox active sites, leading to dramatic enhancement of capacitance to 1020 F g^−1^ at 0.5 A g^−1^. Such a highly alkaline-stable Co_3_O_4_@Co-MOF composite shows significant advantages for application as an electrochemical capacitor energy storage device electrode in terms of enhanced durability and capacitance. The Co_3_O_4_@Co-MOF composite shows a high cycling stability after 5000 cycles with only 3.3% decay at 5 A g^−1^. More remarkably, the as-constructed aqueous/solid-state device showed high specific capacitance, wonderful cycle stability and high energy density. In addition, the as-fabricated solid-state flexible device showed excellent mechanical flexibility and environmental stability. Considering the merits of the facile synthetic method, simple construction and outstanding properties, the Co_3_O_4_@Co-MOF//AC solid-state flexible device opens up bright prospects in portable, flexible and lightweight electronic applications.

## Supplementary Material

nwz137_Supplemental_File
