# Supplementary material for: A highly alkaline-stable metal oxide@metal–organic framework composite for high-performance electrochemical energy storage
Source: Natl Sci Rev. 2019 Sep 12;7(2):305–14. doi: 10.1093/nsr/nwz137 (PMC8288962; doi:10.1093/nsr/nwz137)
Supplement: nwz137_Supplemental_File [file nwz137_supplemental_file.docx]

**Supporting Information**

**A highly alkaline-stable metal oxide@metal-organic framework** **composite for high-performance electrochemical energy storage**

Shasha Zheng,^1^ Qing Li,^1^ Huaiguo Xue,^1^ Huan Pang,^1,^* Qiang Xu^1,2,^*

^1^School of Chemistry and Chemical Engineering, and Institute for Innovative Materials and Energy, Yangzhou University, Yangzhou, 225009, Jiangsu, P. R. China.

^2^AIST-Kyoto University Chemical Energy Materials Open Innovation Laboratory (ChEM-OIL), National Institute of Advanced Industrial Science and Technology (AIST), Yoshida, Sakyo-ku, Kyoto 606-8501, Japan.

E-mail: huanpangchem@hotmail.com; panghuan@yzu.edu.cn; q.xu@aist.go.jp; qxuchem@yzu.edu.cn

Homepage: http://huanpangchem.wix.com/advanced-material

Content

1. Experimental section 2

1.1 Materials 2

1.2 Materials synthesis 2

1.3 Table S1. Experimental parameters for the samples. 3

1.4 Material characterization 3

1.5 Fabrication of the electrodes in a traditional three-electrode system 4

1.6 Fabrication of the aqueous electrochemical energy storage device 4

1.7 Fabrication of the solid-state flexible electrochemical energy storage device 5

2. Calculations 6

3. SEM images of Co-MOF+ptcda, Co-MOF, and Co_3_O_4_@Co-MOF 7

4. SEM images of Co_3_O_4_ 8

5. Optical pictures of Co-MOF+ptcda, Co-MOF and Co_3_O_4_@Co-MOF 9

6. XRD and IR patterns of Co-based nanomaterials 10

7. The structure analysis of Co-MOF 11

8. XPS spectra of the Co 2p 12

9. XPS spectra of the O 1s 13

10. XPS spectra of the C 1s 14

11. BET of the Co-MOF+ptcda, Co-MOF and Co_3_O_4_@Co-MOF 15

12. Pore size distribution of the Co-MOF+ptcda, Co-MOF and Co_3_O_4_@Co-MOF 16

13. TG curve of Co_3_O_4_@Co-MOF 17

14. EDS analysis of Co_3_O_4_@Co-MOF 19

15. Table S2. Co_3_O_4_/Co-MOF mole ratio in Co_3_O_4_@Co-MOF 20

16. SEM images of Co_3_O_4_+Co-MOF 21

17. CV curves at different potentials in a three-electrode cell 22

18. CV curves of sample at different scan rates in a three-electrode cell 23

19. The GCD curves at different current densities in a three-electrode cell 24

20. The GCD curves of Co_3_O_4_@Co-MOF at high current densities 25

21. The electrochemical impedance spectra in a three-electrode cell 26

22. SEM images of Co_3_O_4_@Co-MOF after cycling for 5000 cycles 27

23. TEM images of Co_3_O_4_@Co-MOF after cycling for 5000 cycles 28

24. EDS of Co_3_O_4_@Co-MOF after cycling for 5000 cycles 29

25. Optical and SEM images of Co-MOF after immersion in 3.0 M KOH for 0 h, 24 h, 7 days and 15 days 30

26. XRD patterns of Co-MOF for 0 h and 15 days. 31

27. SEM images of Co-MOF and Co_3_O_4_@Co-MOF after immersion in HNO_3_ solution 32

28. SEM images of Co-MOF and Co_3_O_4_@Co-MOF after immersion in HNO_3_ solution 33

29. XPS spectra of Co_3_O_4_@Co-MOF, Co_3_O_4_@Co-MOF after immersed in 3.0 M KOH for 15 days and after cycling for 5000 cycles 34

30. The GCD curves of activated carbon 35

31. CV curves of the aqueous device with a scan rate at 50 mV s^-1^ at different potentials 36

32. CV curves of the aqueous device at different scan rates 37

33. The GCD curves of the aqueous device at different current densities 38

34. Electrochemical characterization of the aqueous device 39

35. The thickness of the electrode 40

36. The electrochemical impedance spectra of the aqueous device 41

37. CV curves of the solid-state ﬂexible device at different potentials 42

38. CV curves of the solid-state ﬂexible device at different scan rates 43

39. The GCD curves of the solid-state ﬂexible device at different potentials 44

40. The GCD curves of the solid-state ﬂexible device at different current densities 45

41. The electrochemical impedance spectra of the solid-state ﬂexible device 46

42. TEM images of Co_3_O_4_@Co-MOF after 400 bending cycles 47

43. Table S3. Comparison of the obtained materials. 48

44. Table S4. A comparison with previously reported metal oxide nanomaterials. 49

45. Table S5. A comparison with previously reported MOF nanomaterials. 50

46. Table S6. A comparison with previously reported MOF composites. 51

47. Table S7. Comparison of the capacitance value of Co_3_O_4_@Co-MOF at high current densities with previously reported MOF-based materials. 52

48. References 53

1. **Experimental section**
   1. **Materials**

All chemicals, perylene-3,4,9,10-tetracarboxylic dianhydride (ptcda, C_24_H_8_O_6_, 98%), cobalt acetate tetrahydrate (Co(CH_3_COO)_2_·4H_2_O), and sodium hydroxide (NaOH, 96%), were purchased from Shanghai Sinopharm Chemical Reagent Co. and used without further purification. All aqueous solutions were freshly prepared with deionized water (18 MΩ cm).

- 1. **Materials synthesis**

**Synthesis of Co-MOF+ptcda.** In a typical synthesis, ptcda (0.2 mmol) and NaOH (0.4 mmol) were dissolved in 12.5 mL deionized water with stirring at room temperature, and Co(AC)_2_·4H_2_O (0.2 mmol) was dissolved in 22.5 mL of deionized water. Subsequently, the ptcda solution was added dropwise to metal acetate solution with magnetic stirring. The mixture was transformed into a Teflon-lined stainless steel autoclave. The autoclave was maintained at 100 °C for 12 h, and then naturally cooled to room temperature. The resulting precipitate was thoroughly washed several times with deionized water and alcohol, respectively.

**Synthesis of Co-MOF.** Co-MOF was synthesised as described above, using ptcda and NaOH with a mole ratio of 1 : 4.

**Synthesis of Co_3_O_4_@Co-MOF.** Co_3_O_4_@Co-MOF was synthesised using a similar procedure to that described above, except for the use of ptcda and NaOH with a mole ratio of 1 : 6.

**Synthesis of Co_3_O_4_.** The preparation process of Co_3_O_4_ was same as that of Co_3_O_4_@Co-MOF, except that ptcda is removed.

**Synthesis of Co_3_O_4_+Co-MOF.** Co_3_O_4_ nanocubes and Co-MOF with a mole ratio of 1.5 : 1 (mass ratio of 1 : 4) were dispersed into 20 mL ethanol, and then was stirred for 30 min to form a black mixture. The obtained mixture was filtered, and dried in air naturally.

- 1. **Table S1. Experimental parameters for the samples.**

| **Sample** | **Chemical agents** | | **Hydrothermal** | |
| --- | --- | --- | --- | --- |
|  | **Co(AC)_2_·4H_2_O : ptcda : NaOH (mole ratio)** | **H_2_O** | **T(^o^C)** | **Time** |
| Co-MOF+ptcda | 1 : 1 : 2 | 35 mL | 100 | 12 h |
| Co-MOF | 1 : 1 : 4 | 35 mL | 100 | 12 h |
| Co_3_O_4_@Co-MOF | 1 : 1 : 6 | 35 mL | 100 | 12 h |
| Co_3_O_4_ | 1 : 0 : 6 | 35 mL | 100 | 12 h |

**Table S1** show that ptcda : NaOH=1 : 2 contained the incomplete reaction ptcda. However, Co-MOF were successfully synthesized when the mole ratio of ptcda and NaOH is 1 : 4. Subsequently, by adjusting the mole ratio to 1 : 6, Co_3_O_4_@Co-MOF were successfully synthesized. According to the theoretical complete reaction mole ratio of ptcda and NaOH to 1 : 4, uniformity Co-MOF were successfully obtained. As the mole ratio of ptcda and NaOH increases to 1 : 6, excess OH^-^ in the solution forms Co(OH)_2_ with Co(II) exposed on the surface of Co-MOF, and Co(OH)_2_ decomposes into Co_3_O_4_ under hydrothermal conditions.

- 1. **Material characterization**

The morphological features were characterized by field emission scanning electron microscopy (FESEM, Zeiss-Supra55), high resolution transmission electron microscopy (HRTEM, Tecnai G2 F30 S-TWIN), and energy dispersive X-ray spectrometry (EDS) mapping. X-ray diffraction (XRD) patterns were examined on a Bruker D8 Advanced X-ray Diffractometer (Cu-K*α* radiation: *λ* = 0.15406 nm). The chemical states were measured using an Axis Ultra X-ray photoelectron spectroscope (XPS, Kratos Analytical Ltd., UK) equipped with a standard monochromatic Al-K*α* source (hv = 1486.6 eV). Fourier transform infrared (FTIR) transmission spectra were obtained on a BRUKER-EQUINOX-55 IR spectrophotometer. N_2_ adsorption-desorption measurements were performed on Quantachrome Instruments, Autosorb IQ3. The thermogravimetric analysis (TGA) was performed under air atmosphere with a heating rate of 5 ^o^C/min by using a Pyris 1 TGA thermogravimetric analyzer.

- 1. **Fabrication of the electrodes in a traditional three-electrode system**

Electrochemical performance was conducted by a CHI 660E instrument in a traditional three-electrode system. A Hg/HgO electrode and platinum electrode were chosen as reference and counter electrode, respectively. The electrolyte was 3.0 M KOH aqueous solution. The working electrode was prepared by grinding the mixture of active materials, acetylene black, and polytetrafluoroethylene (PTFE) with a weight ratio of 80:15:5, and coating the mixture on a 1 cm × 5 cm nickel foam. The additive was a certain amount of isopropyl alcohol when grinding and the painted size was about 1 cm^2^. In addition, we pressed the nickel foam to a thin foil with a pressure of 10.0 MPa. The typical mass loading of the active material was ≈3 mg.

- 1. **Fabrication of the aqueous electrochemical energy storage device**

Aqueous electrochemical energy storage devices were assembled by employing the MOF-based materials as positive electrode and activated carbon as negative electrode. The mass loading for the negative electrode was determined by balancing the charges stored in each electrode. Generally, the charges stored by positive and negative electrodes can be determined by q_+_ = C_+_ × △E_+_ × m_+_ and q_-_ = C_-_ × △E_-_ × m_-_, where C_+_, C_-_ represent the specific capacitance of positive electrode and negative electrodes (F g^-1^), respectively; △E is the potential range (V); m_+_, m_-_ is the weight of the active material in positive electrode and negative electrode (g), respectively; The charges are balanced by the equation of q_+_ = q_-_, where q_+_ and q_-_ represent the charges stored in the positive and negative electrodes, respectively. Therefore, m_+_/m_-_ = C_-_ × △E_-_/C_+_ × △E_+_. The specific capacitance of purchased activated carbon electrode was 168 F g^-1^ when the current density was 1 A g^-1^. Thus, the mass ratio between the positive and negative electrodes was set at 1:4 in the as-assembled device. The electrochemical performance of the devices was measured at room temperature in a two-electrode electrochemical full cell. The electrolyte was 3.0 M KOH aqueous solution.

- 1. **Fabrication of the solid-state flexible electrochemical energy storage device**

The positive and negative electrodes were prepared in the same way as the electrodes of aqueous device. The PVA/KOH gel electrolyte was prepared as follows: 1.52 g PVA were added to 15 mL deionized water and the as-obtained solution was heated to 75 °C for 30 min, then 5 mL KOH aqueous solution was added dropwise into the gel solution under stirring. The positive and negative electrodes were placed on different sides of the PET substrate, and then coated with the gel solution covering the active materials. After the excess water was vaporized, the positive and negative electrodes including electrolyte sandwiched between two pieces of PET substrates. Then, the all-solid-state ﬂexible device was fabricated.

1. **Calculations**

The mass-specific capacitance (*C/F g^-1^*) of the device can also be calculated using：

*C=Q / (m×△V)= ∫Idt / (m×△V)= I×t_discharge_ / (m×△V)*  (1)

where *m* is the mass of the activated materials, *I* is the discharge current, *t_discharge_* is

discharge time, and *△V* is the potential drop during discharge.

The area-specific capacitance (*C/mF cm^-2^*) of the device can also be calculated using：

*C=Q / (A×△V)= ∫Idt / ( A×△V)= I×t_discharge_ / ( A×△V)*  (2)

where *A* is the surface area of the device, *I* is the discharge current, *t_discharge_* is

discharge time, and *△V* is the potential drop during discharge.

The energy density and power density of the device can be obtained from：

*E=0.5C×V^2^*  (3)

*P=E / t_discharge_*  (4)

where *V* represents the operating voltage.

1. **SEM images of Co-MOF+ptcda, Co-MOF, and Co_3_O_4_@Co-MOF**

**
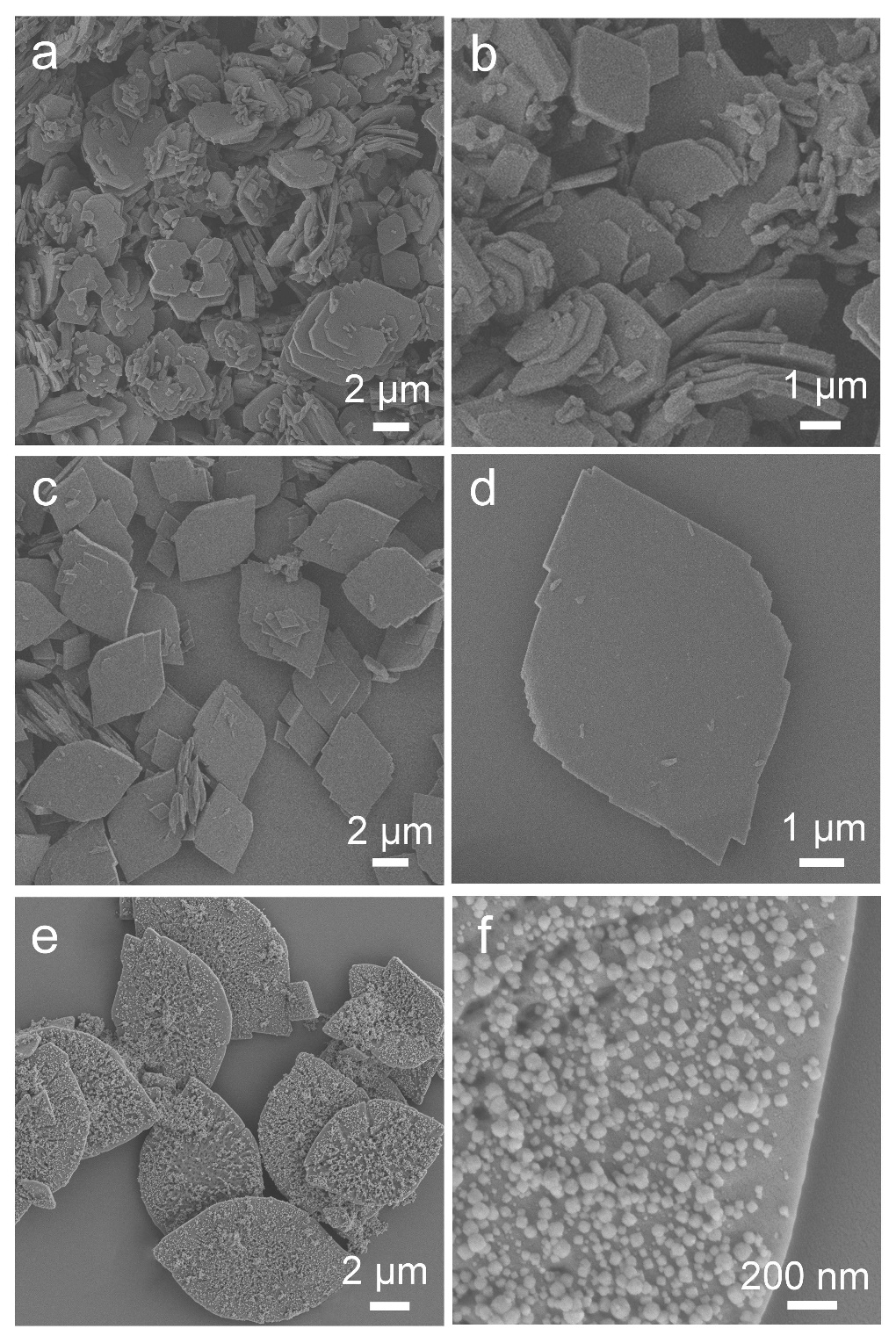
**

**Figure S1.** SEM images of (a,b) Co-MOF+ptcda, (c,d) Co-MOF, and (e,f) Co_3_O_4_@Co-MOF.

1. **SEM images of Co_3_O_4_**

**
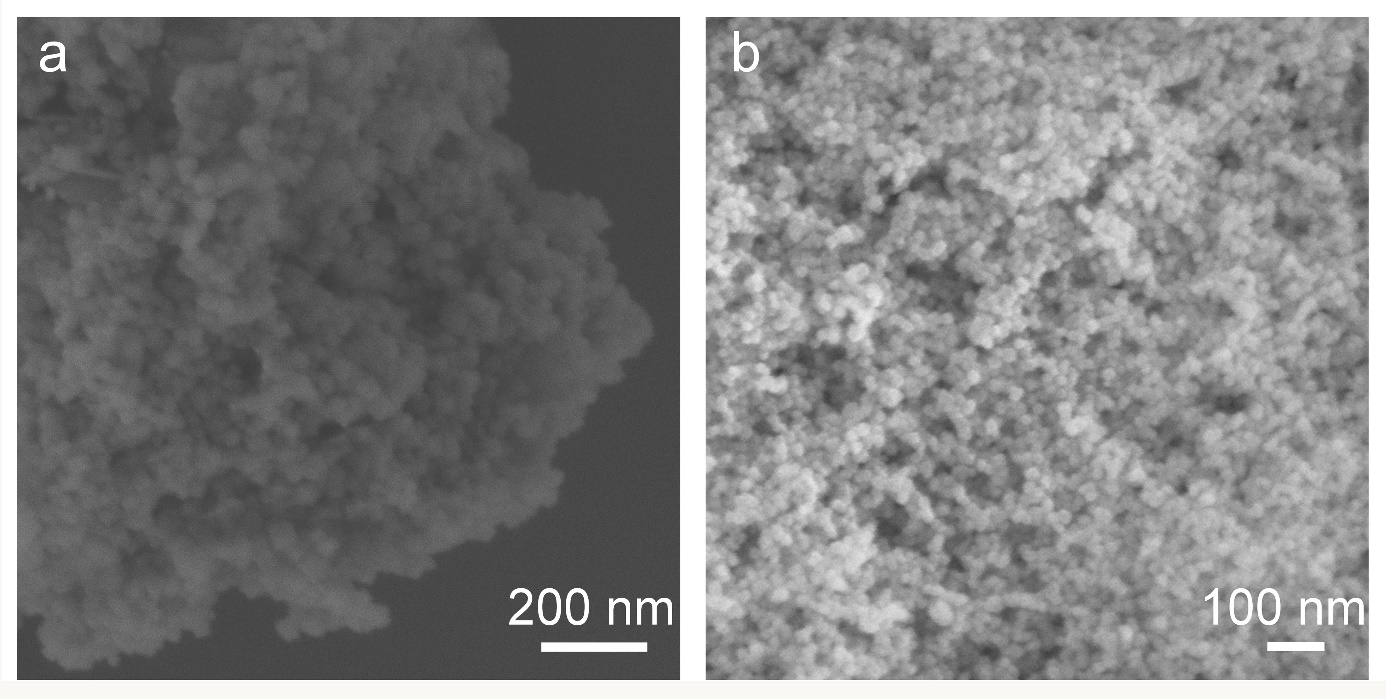
**

**Figure S2.** SEM images of Co_3_O_4_.

1. **Optical pictures of** **Co-MOF+ptcda, Co-MOF and Co_3_O_4_@Co-MOF**

**
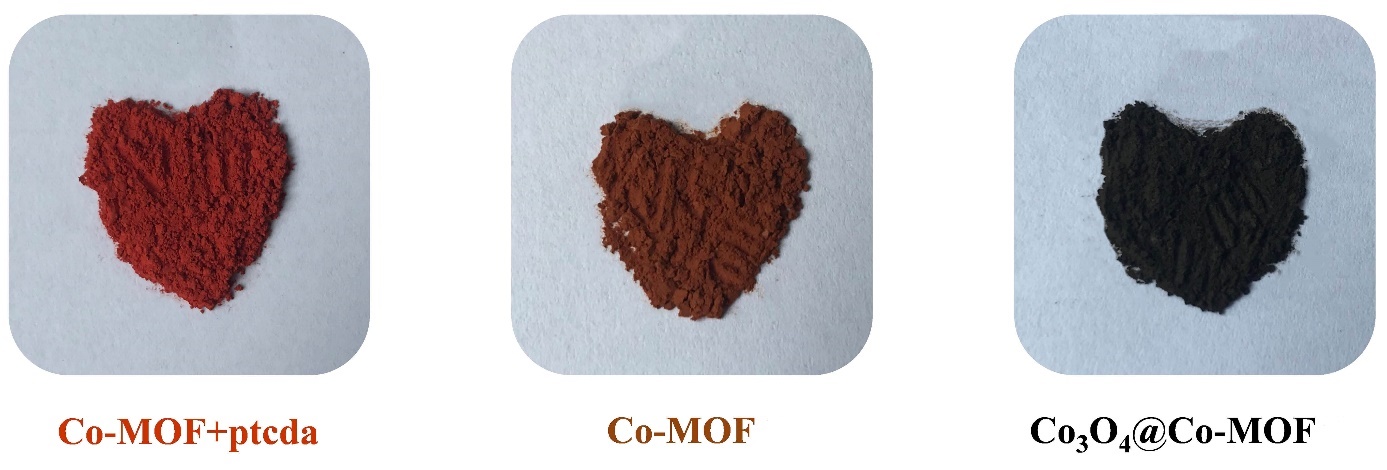
**

**Figure S3.** Optical pictures of Co-MOF+ptcda, Co-MOF and Co_3_O_4_@Co-MOF.

1. **XRD and IR patterns of Co-based nanomaterials**

**
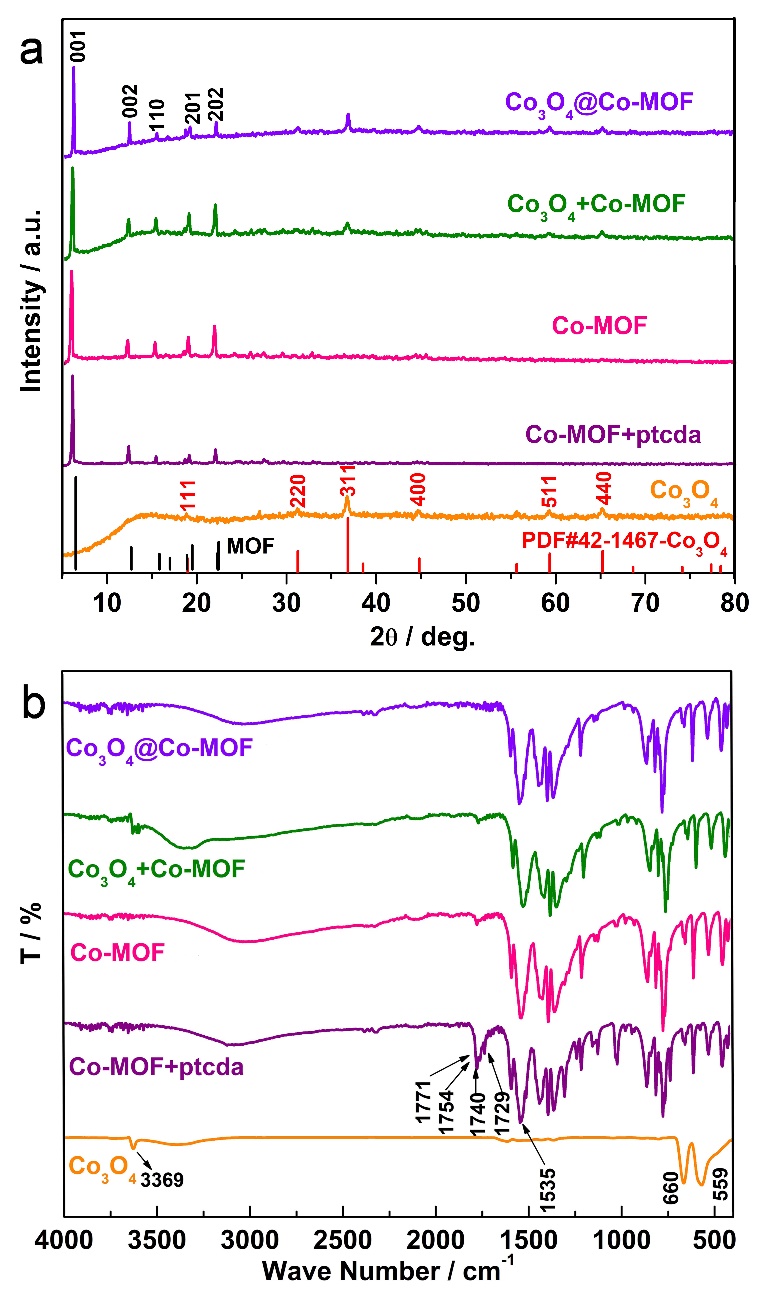
**

**Figure S4.** XRD (a) and IR (b) patterns of Co_3_O_4_, Co-MOF+ptcda, Co-MOF, Co_3_O_4_+Co-MOF, Co_3_O_4_@Co-MOF.

The Fourier transform infrared (FTIR) spectra of the Co-MOF-based materials are displayed in **Figure S4**b. After the reaction of Co^2+^ with ptcda, the typical C=O stretching vibrations (1772, 1755, 1741, and 1730 cm^-1^) disappear, and a new strong band at 1535 cm^-1^ appears, corresponding to the anti-symmetric stretching vibration of –COO–, confirming the successful coordination of the carboxyl groups to Co^2+^. Although C=O stretching vibrations still exist for the Co-MOF+ptcda, a strong band at 1535 cm^-1^ can be observed, mainly because of the incomplete reaction of ptcda.

1. **The structure analysis of Co-MOF**

**
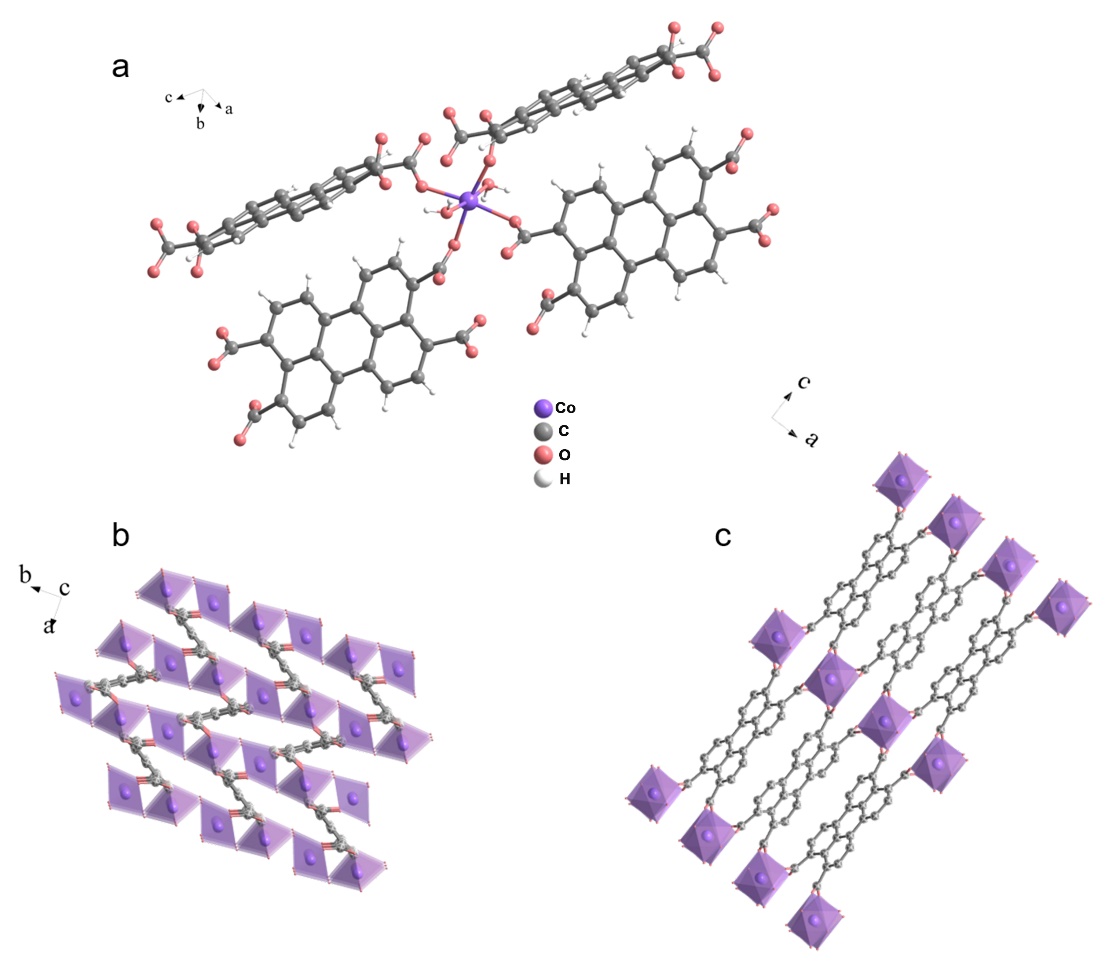
**

**Figure S5.** The structure analysis of Co-MOF: (a) coordination environments of Co(II) and (b and c) model of 3D framework structure of Co-MOF (the coordination environments around Co(II) are shown as light purple octahedra).

The structure analysis of Co-MOF are displayed in **Figure S5**. One Co atom, half ptcda ligand and one coordinated H_2_O molecule compose the building unit. The central Co atom is surrounded by six O atoms to form an octahedral chain interlinked by H_2_O molecules along the b direction, forming a wavy -O-Co-O-Co- backbone. The [CoO_6_] octahedral local structure makes the perylene core interlinked to form a 3D open framework with wavy-layered structure. The coordination between organic ligands and metal ions creates a controllable interlayer spacing, which is favorable for K^+^ transportation between the organic layers. For each organic ligand, all eight carboxylate oxygen atoms are extended outward from the perylene layer and coordinated to different Co atoms.

1. **XPS spectra of the Co 2p**

**
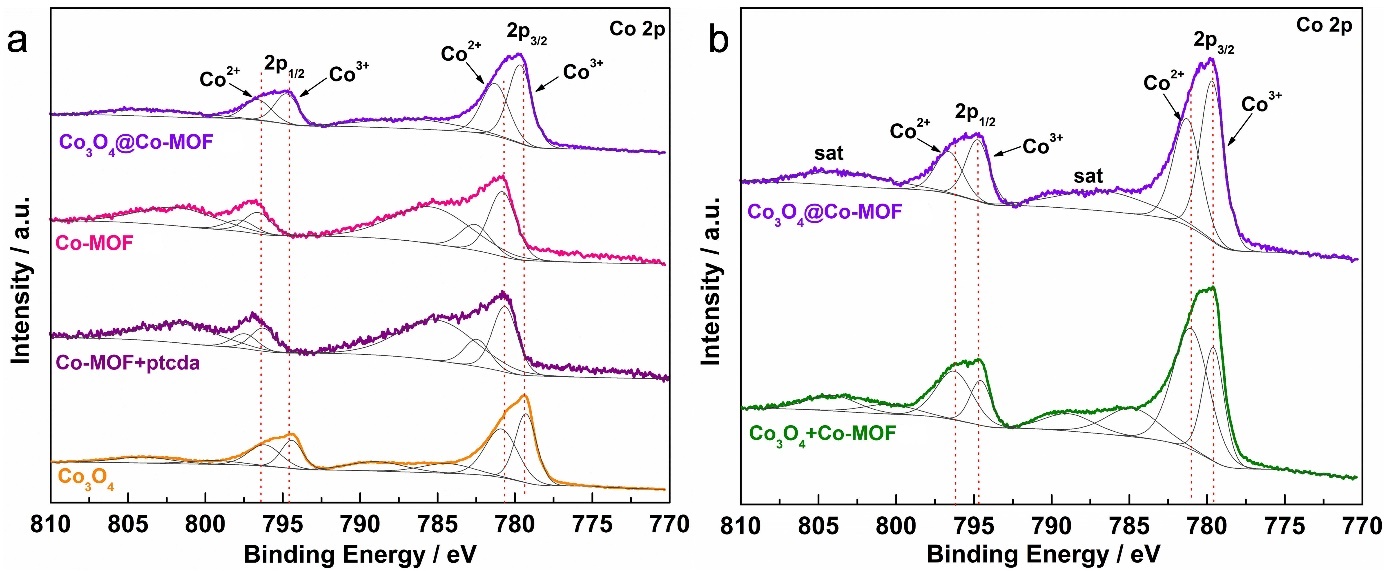
**

**Figure S6.** XPS spectra of a) the Co 2p of the Co-MOF+ptcda, Co-MOF, Co_3_O_4_, Co_3_O_4_@Co-MOF, and b) the Co 2p of Co_3_O_4_+Co-MOF, Co_3_O_4_@Co-MOF.

1. **XPS spectra of the O 1s**

**
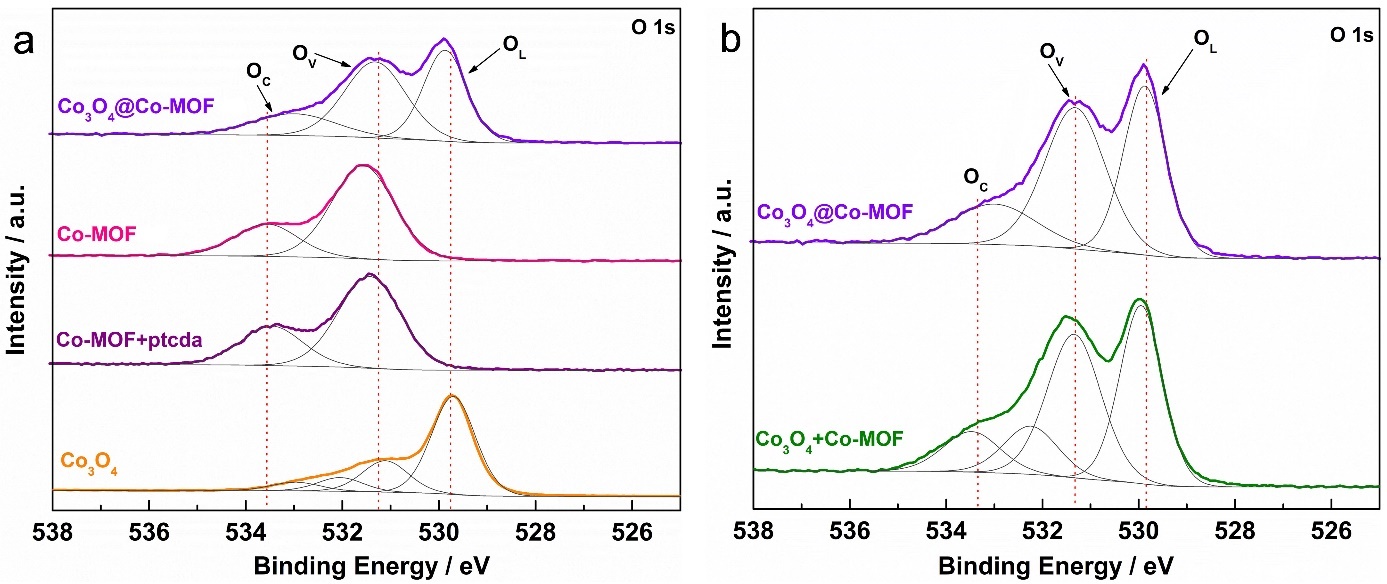
**

**Figure S7.** XPS spectra of a) the O 1s of the Co-MOF+ptcda, Co-MOF, Co_3_O_4_, Co_3_O_4_@Co-MOF, and b) the O 1s of Co_3_O_4_+Co-MOF, Co_3_O_4_@Co-MOF.

**Figure S7** shows the XPS spectra of O 1s, which could be fitted by three peaks at binding energies of around 529.7, 531.2, and 533.4 eV, attributing to the lattice oxygen (O_L_), oxygen vacancy (O_V_) and chemisorbed oxygen species (O_C_), respectively. What is more, the O_L_ fitting peaks of Co_3_O_4_, Co_3_O_4_+Co-MOF and Co_3_O_4_@Co-MOF are also observed, which means that Co_3_O_4_ exists in this system.

1. **XPS spectra of the C 1s**

**
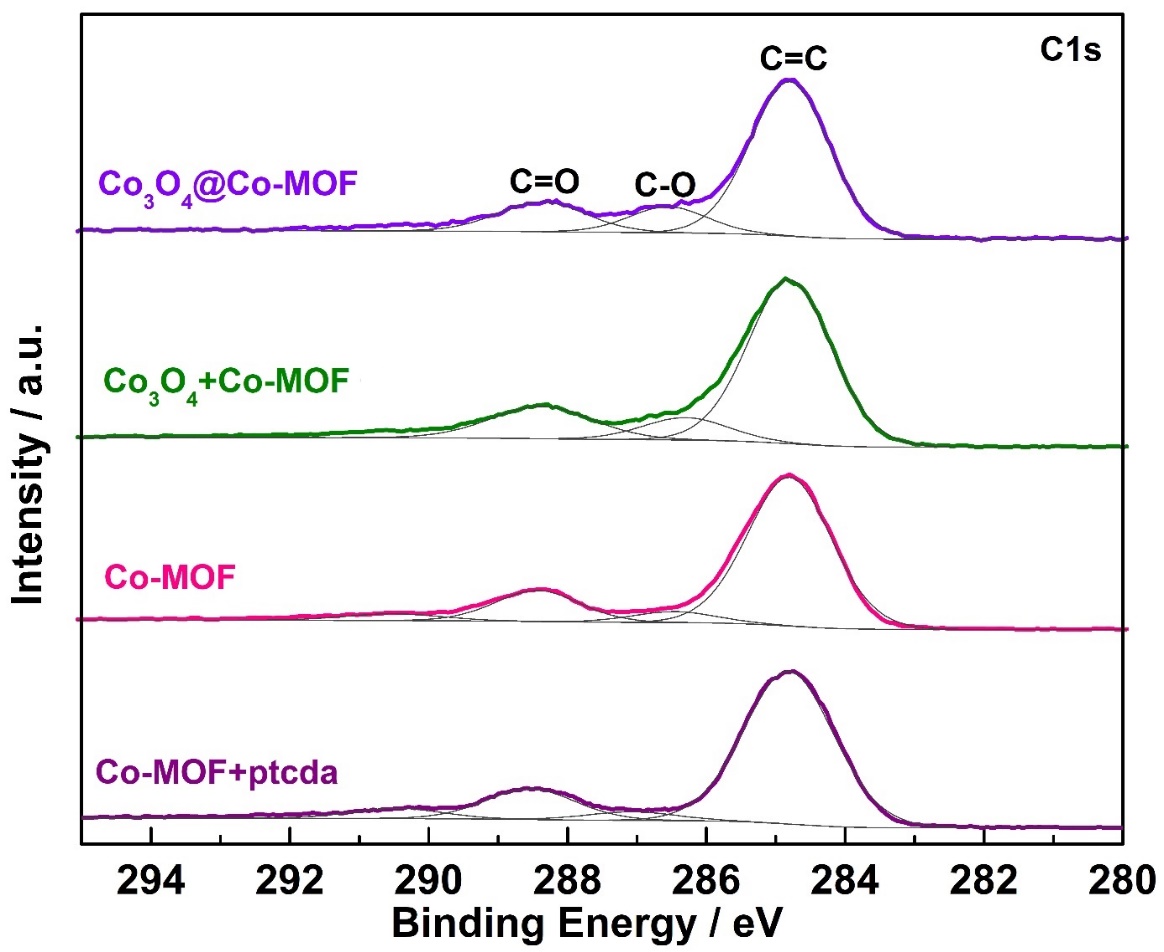
**

**Figure S8.** XPS spectra of the C 1s of the Co-MOF+ptcda, Co-MOF, Co_3_O_4_+Co-MOF, Co_3_O_4_@Co-MOF.

The XPS spectra of C 1s was shown in **Figure S8,** the possible presence of C=O (288.1 eV), C-O (286.2 eV), and C=C (284.6 eV) species in the C 1s spectrum.

1. **BET of the** **Co-MOF+ptcda, Co-MOF and Co_3_O_4_@Co-MOF**

**
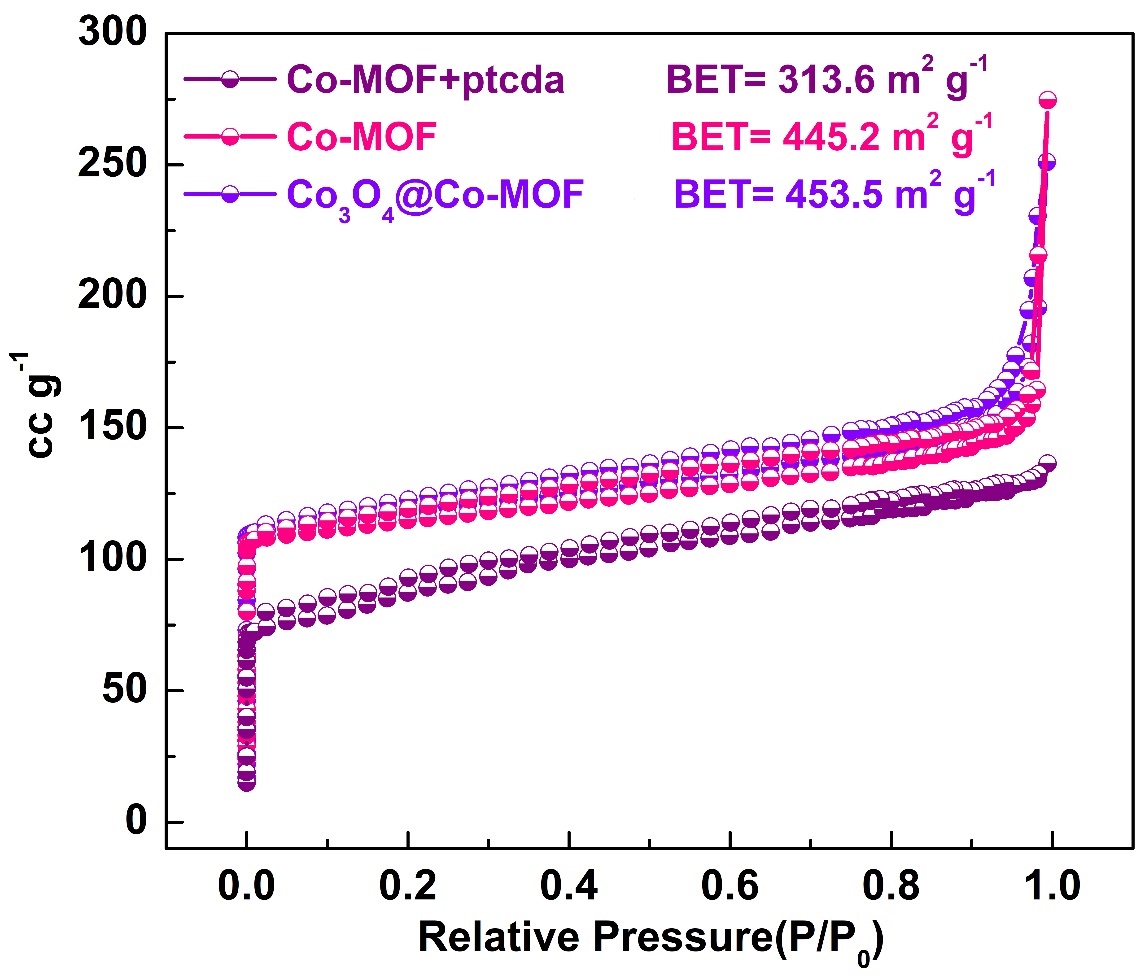
**

**Figure S9.** N_2_ adsorption-desorption isotherms (77 K) of Co-MOF+ptcda, Co-MOF and Co_3_O_4_@Co-MOF.

The porosity of the samples was examined using N_2_ adsorption-desorption isotherms at 77 K. According to the IUPAC classification, the Co_3_O_4_@Co-MOF composite and Co-MOF show a combination of types I and IV isotherms; Co-MOF+ptcda shows type I isotherm curves.

1. **Pore size distribution of the Co-MOF+ptcda, Co-MOF and Co_3_O_4_@Co-MOF**

**
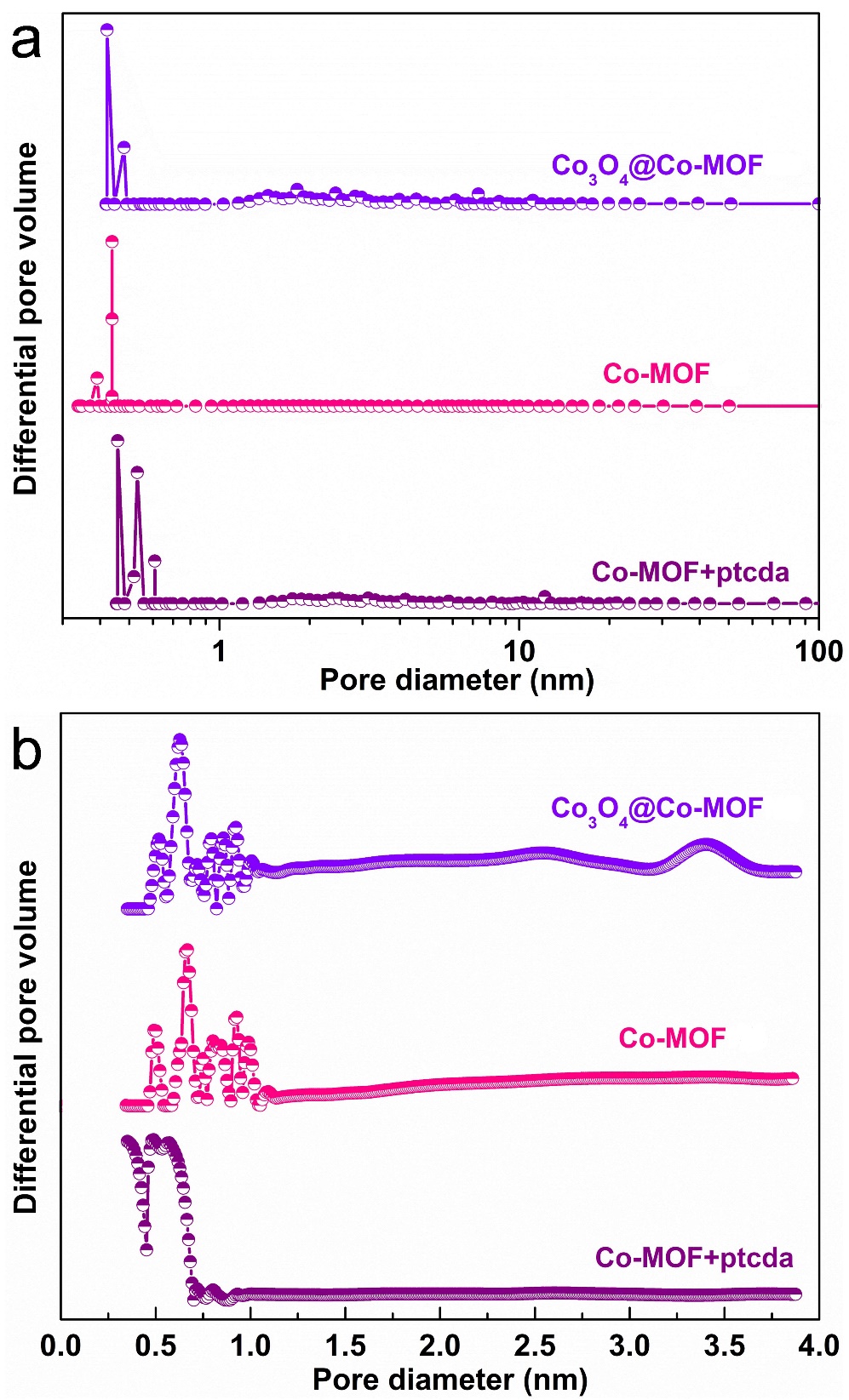
**

**Figure S10.** The pore size distribution of the Co-MOF+ptcda, Co-MOF and Co_3_O_4_@Co-MOF for the BJH adsorption branch (a) and SF method (b), showing the co-existence of micropores and mesopores in the Co-MOF+ptcda, Co-MOF and Co_3_O_4_@Co-MOF.

1. **TG curve of Co_3_O_4_@Co-MOF**

**
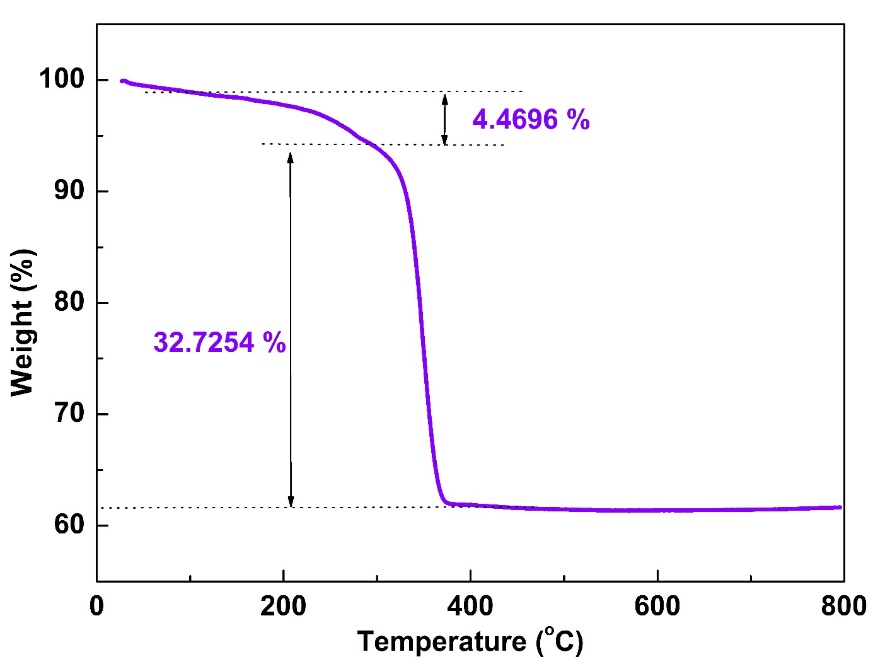
**

**Figure S11.** TG curve of Co_3_O_4_@Co-MOF.

The mass ratio of Co_3_O_4_/Co-MOF in Co_3_O_4_@Co-MOF composites was measured by TG curve. Co_3_O_4_@Co-MOF shows very high thermal stability up to more than 300 ^o^C. According to the TG analysis (**Figure S11**), Co_3_O_4_@Co-MOF has two consecutive weight-loss steps, at 150 and 310 ^o^C, corresponding to the release of the coordinated water molecules (4.4696 wt%) and the loss of organic molecules (32.7254 wt%) due to decarboxylation of the ptcda ligand, respectively. The chemical reaction equations of Co_3_O_4_@Co-MOF for two weight-loss step are as follows:

3Co_2_(C_12_H_4_O_3_)_2_·2H_2_O → 3Co_2_(C_12_H_4_O_3_)_2_ + 6H_2_O (1)

*M* *M_a_* = 546.2137 *M_b_* = 510.1831 *M_c_* = 18.0153

*m* *m_a_ m_b_ m_c_*

*n* *n_a_ n_b_ n_c_*

3Co_2_(C_12_H_4_O_3_)_2_ + 76O_2_ → 2Co_3_O_4_ + 72CO_2_ + 13H_2_O (2)

*M* *M_b_* = 510.1831 *M_d_* = 240.7972

*m* *m_b_ m_d_ m_e_*  *m_f_*

*n* *n_b_ n_d_ n_e_*  *n_f_*

**The known parameters:**

Sample quality before testing: *m_0_* = 1.786 mg; coordinated water molecules, the ptcda ligand, and Co_3_O_4_ (including Co-MOF decomposition and Co_3_O_4_ from Co_3_O_4_@Co-MOF composites, *m_1_*) of weight percentage was 4.4696, 32.7254, and 61.6333 wt%, respectively.

*n_c_* = *m_c_* */M_c_* = *m_0_* × 4.4696% /*M_c_* = 0.004435 mol

*n_b_* = *n_a_* = 3*/*6 × *n_c_* = 0.0022174 *m_b_* = *n_b_* × *M_b_* = 1.13128 mg

*m_e_* + *m_f_*  = *m_b_* × 32.7254% = 0.370216 mg

Co_3_O_4_ (Co-MOF decomposition):

*m_d_* = *m_b_* – (*m_e_* + *m_f_*) = 0.76106 mg

Co_3_O_4_ (Co-MOF decomposition and Co_3_O_4_ from Co_3_O_4_@Co-MOF composites):

*m_1_* = *m_0_* × 61.6333% = 1.10077 mg

Co_3_O_4_ (from Co_3_O_4_@Co-MOF composites):

*m_Co3O4_* = *m_1_* – *m_d_* = 0.33971 mg

Co-MOF (from Co_3_O_4_@Co-MOF composites):

*m_a_* = *n_a_* × *m_a_* = 1.21118 mg

The mass/mole ratio of Co_3_O_4_/Co-MOF in Co_3_O_4_@Co-MOF composites:

*m_a_* : *m_Co3O4_* _=_ 4 : 1 *n_a_* : *n_Co3O4_* _=_ 1 : 1.5

1. **EDS analysis of Co_3_O_4_@Co-MOF**

**
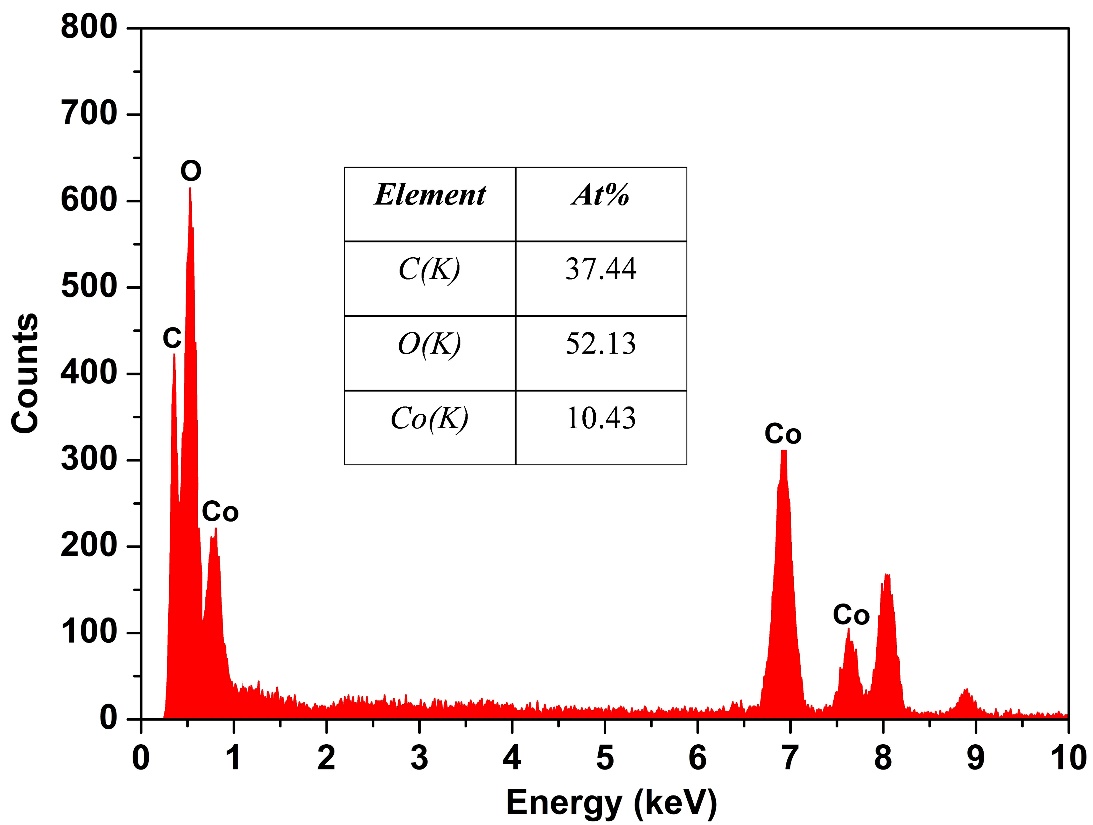
**

**Figure S12.** EDS analysis of Co_3_O_4_@Co-MOF.

1. **Table S2. Co_3_O_4_/Co-MOF mole ratio in Co_3_O_4_@Co-MOF**

Table S2 Co_3_O_4_/Co-MOF mole ratio in Co_3_O_4_@Co-MOF

| data sources | At % | | | Co_3_O_4_/Co-MOF mole ratio  in Co_3_O_4_@Co-MOF |
| --- | --- | --- | --- | --- |
|  | C | O | Co |  |
| XPS | 38.13 | 50.52 | 11.35 | 1.60 : 1 |
| EDS | 37.44 | 52.13 | 10.43 | 1.55 : 1 |
| TG | — | — | — | 1.50 : 1 |

1. **SEM images of Co_3_O_4_+Co-MOF**

**
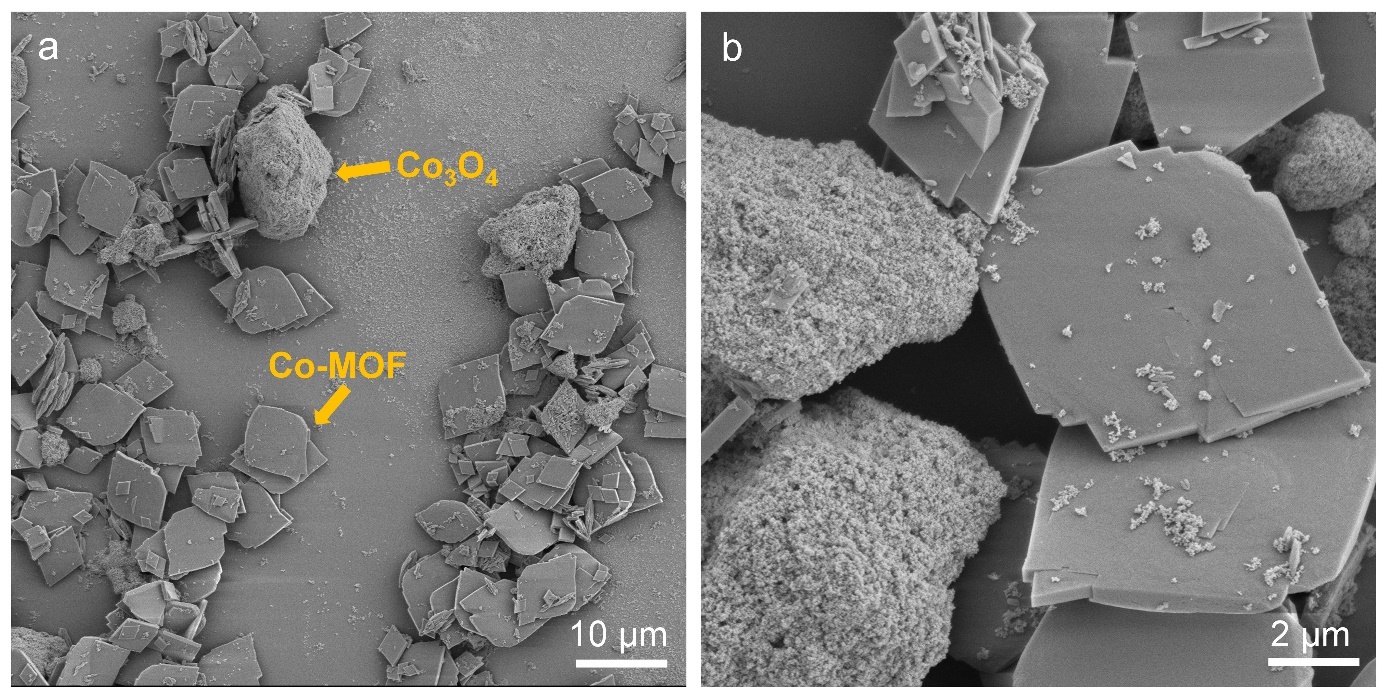
**

**Figure S13**. SEM images of Co_3_O_4_+Co-MOF.

1. **CV curves at different potentials in a three-electrode cell**

**
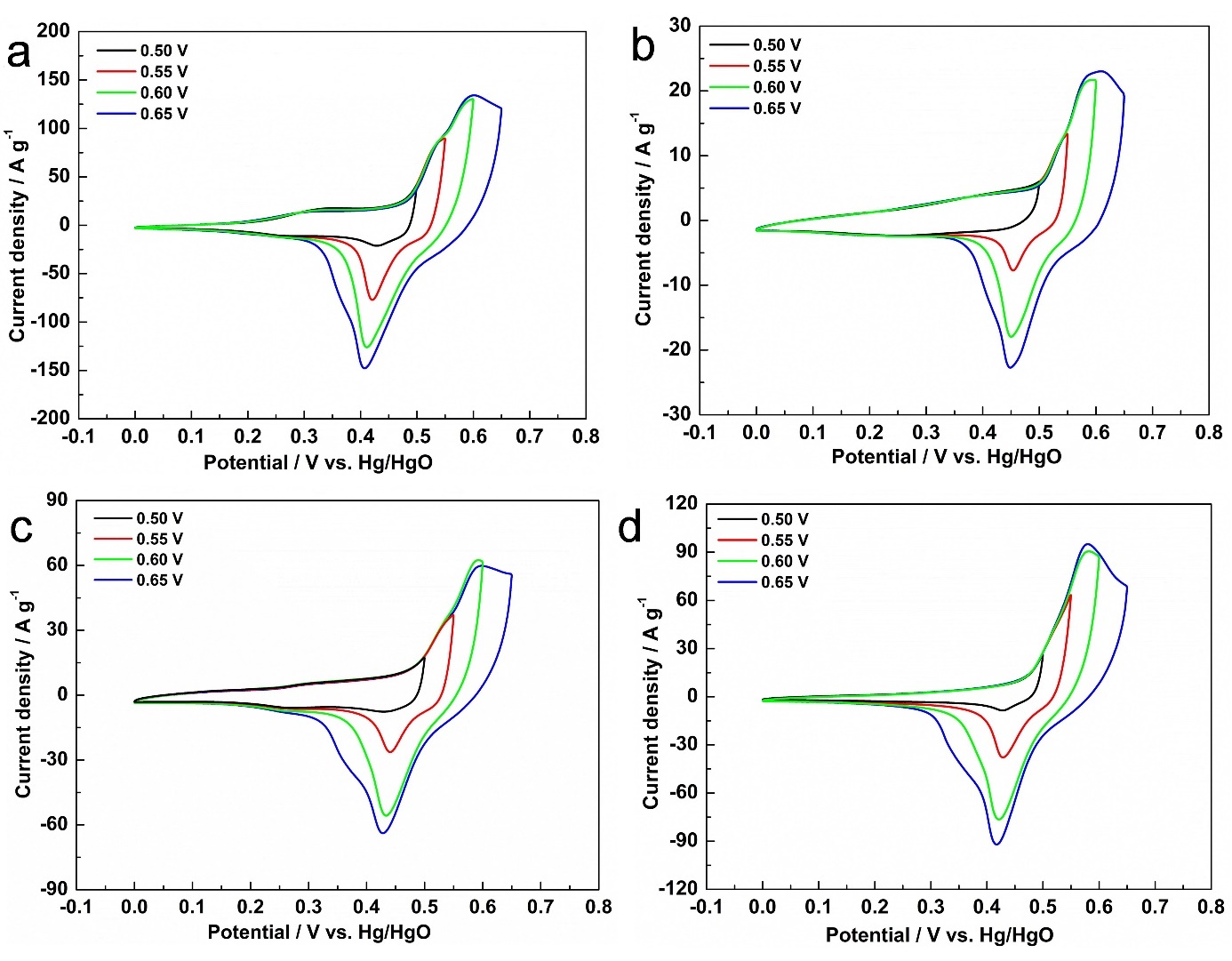
**

**Figure S14.** CV curves with a scan rate at 50 mV s^-1^ of sample in a three-electrode cell in 3.0 M KOH aqueous solution at different potentials: a) Co_3_O_4_@Co-MOF, b) Co-MOF, c) Co_3_O_4_, and d) Co_3_O_4_+Co-MOF.

1. **CV curves of sample at different scan rates in a three-electrode cell**

**
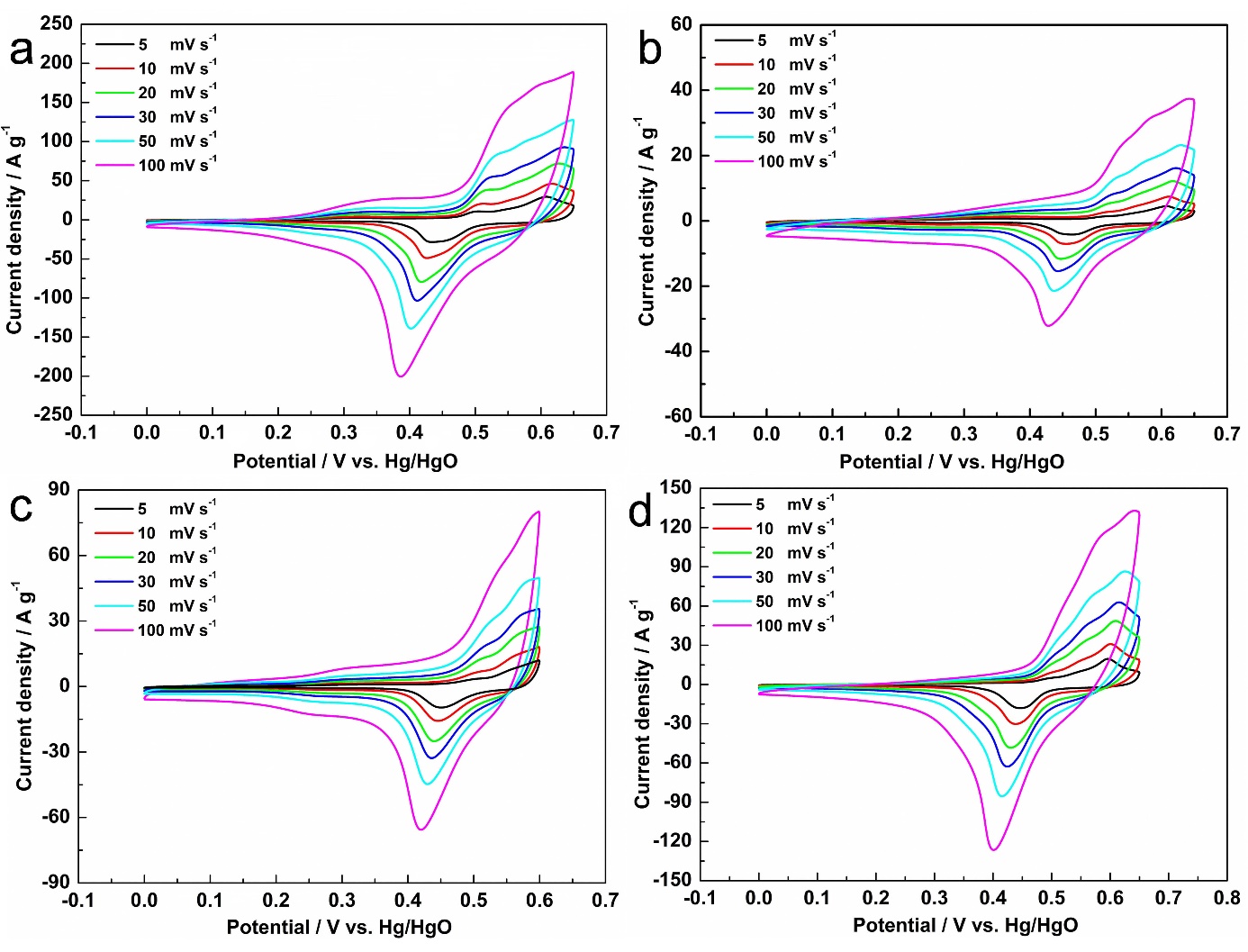
**

**Figure S15.** CV curves of sample in a three-electrode cell in 3.0 M KOH aqueous solution at different scan rates: a) Co_3_O_4_@Co-MOF, b) Co-MOF, c) Co_3_O_4_, and d) Co_3_O_4_+Co-MOF.

With increasing scan rate, the current response increased accordingly. Interestingly, even at a scan rate as high as 100 mV s^-1^, the peak shapes of the CV curves are not distorted to any degree, reflecting the good rate properties.

1. **The GCD curves at different current densities in a three-electrode cell**

**
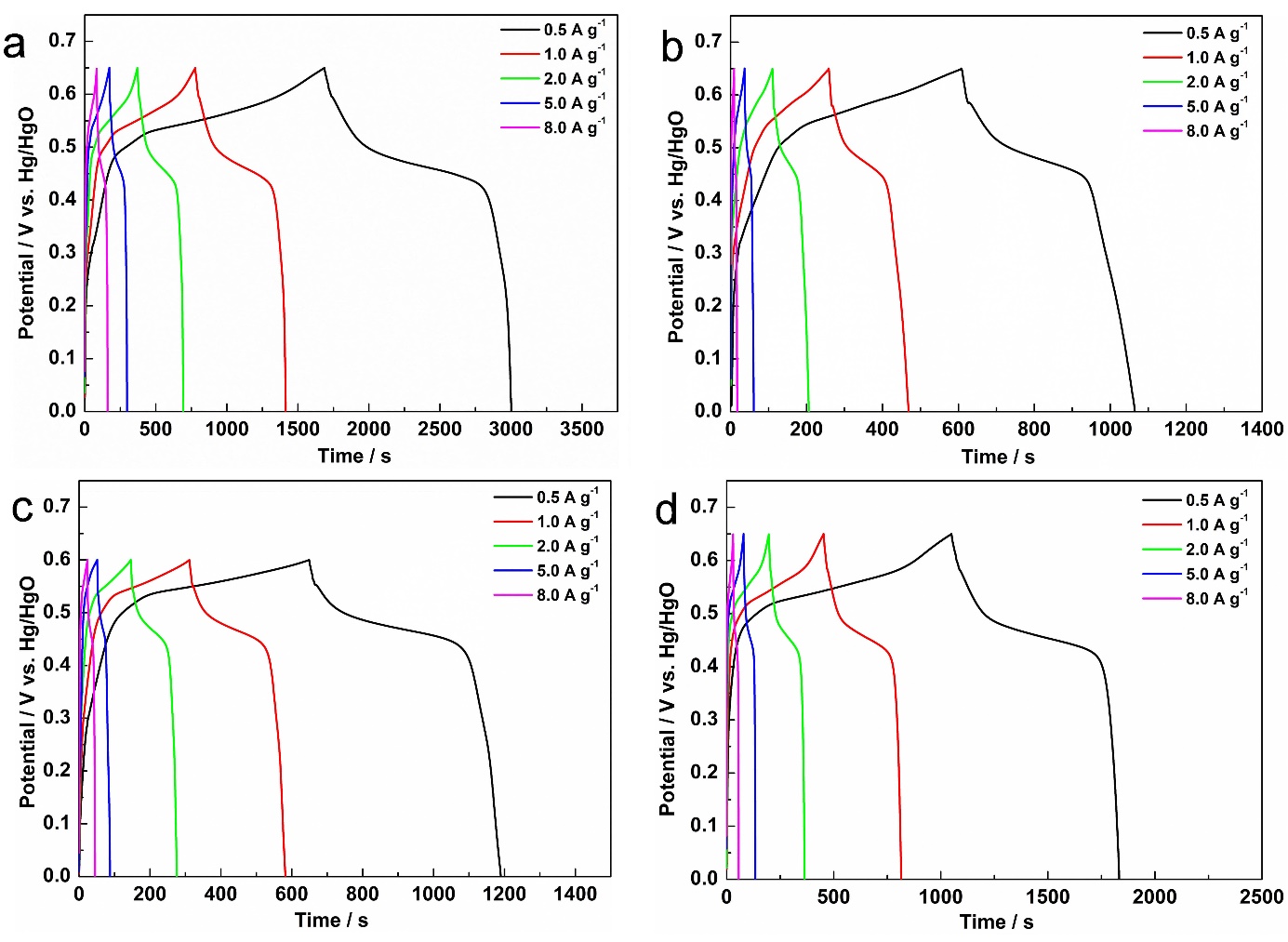
**

**Figure S16.** The GCD curves of sample in a three-electrode cell in 3.0 M KOH aqueous solution at different current densities: a) Co_3_O_4_@Co-MOF, b) Co-MOF, c) Co_3_O_4_, and d) Co_3_O_4_+Co-MOF.

1. **The GCD curves of Co_3_O_4_@Co-MOF at high current densities**

**
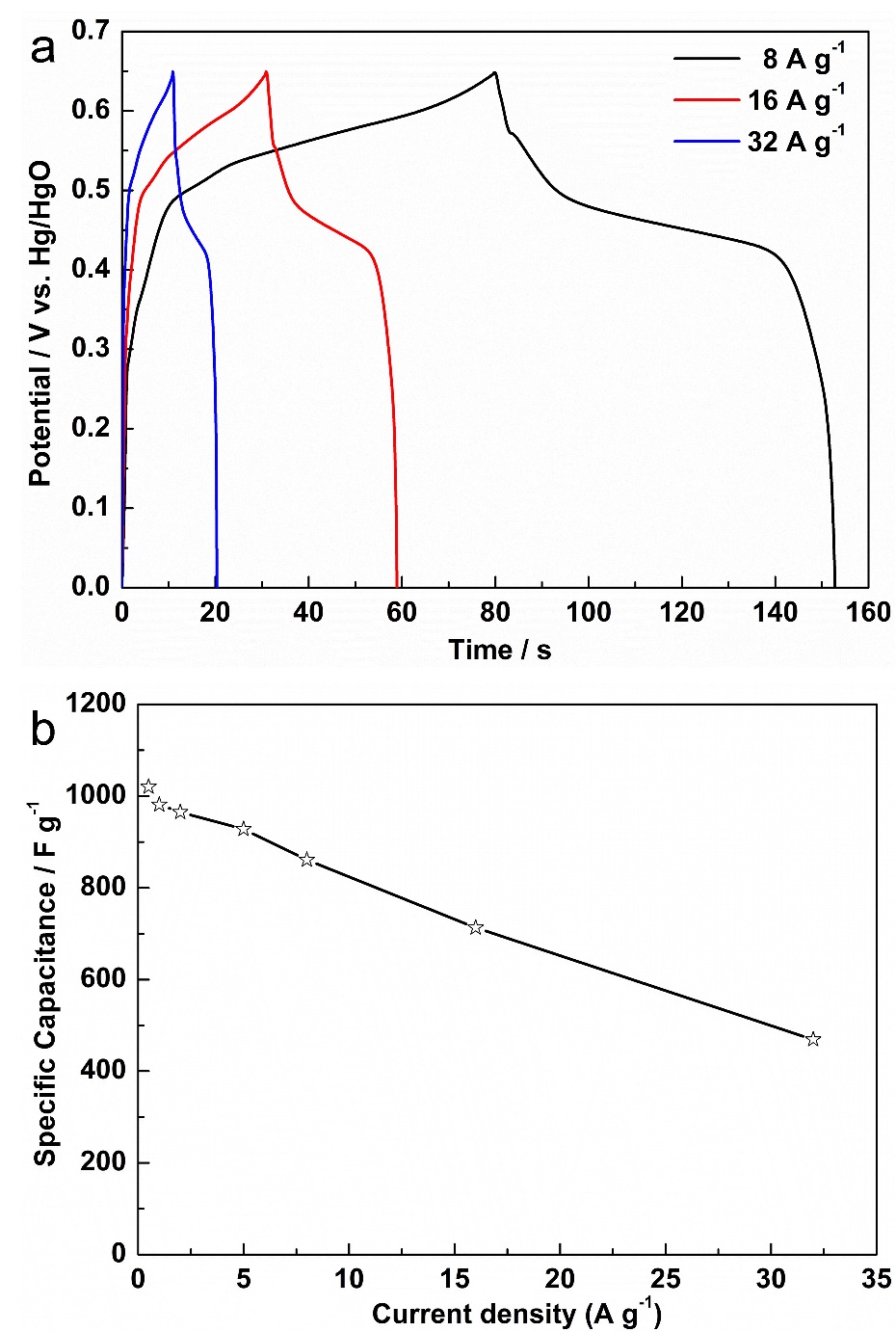
**

**Figure S17.** a) The GCD curves of Co_3_O_4_@Co-MOF in a three-electrode cell at high current densities, b) specific capacitance change vs. current density.

1. **The electrochemical impedance spectra in a three-electrode cell**

**
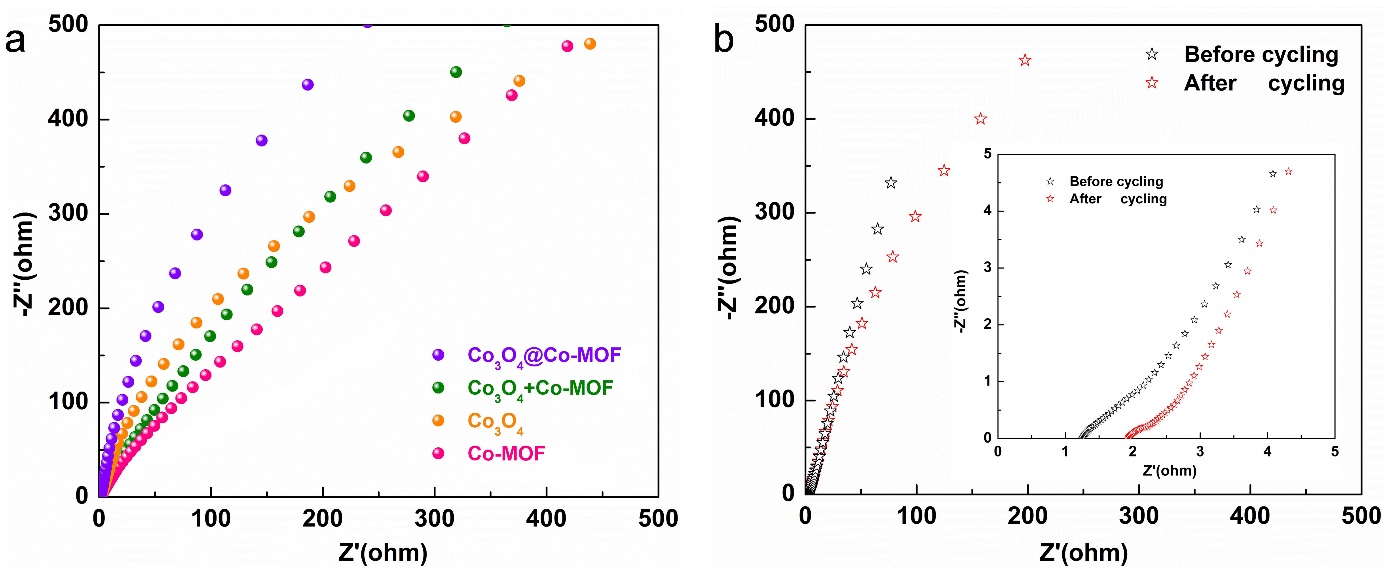
**

**Figure S18.** a) The electrochemical impedance spectra of the sample in a three-electrode cell, and b) before and after cycling for 5000 cycles.

1. **SEM images of Co_3_O_4_@Co-MOF after cycling for 5000 cycles**

**
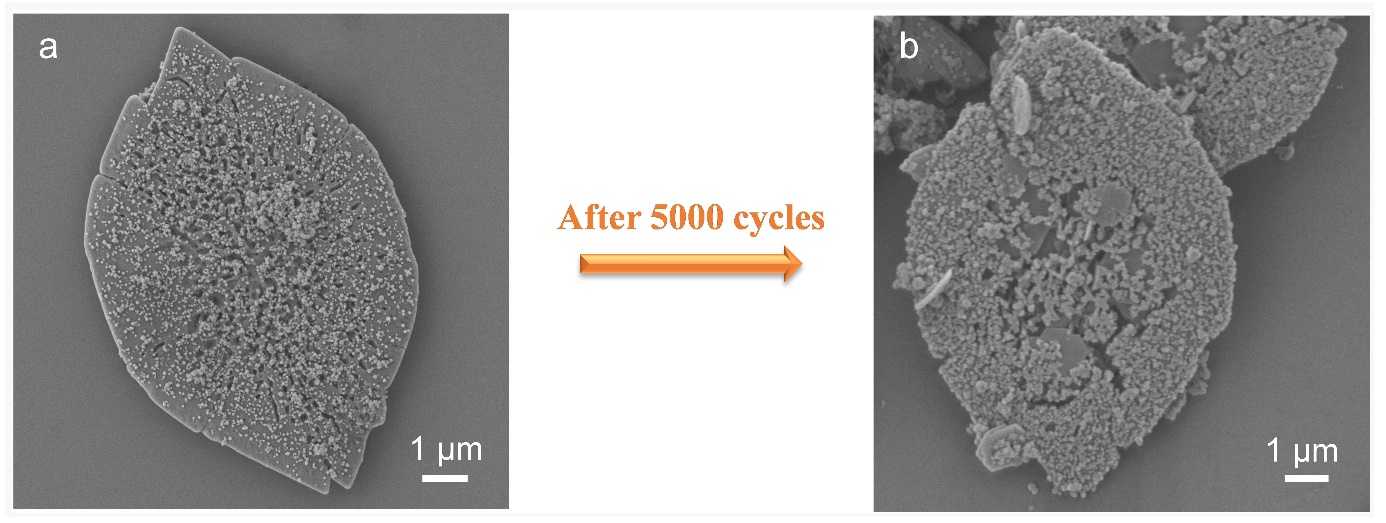
**

**Figure S19.** a) SEM images of Co_3_O_4_@Co-MOF, and b) after cycling for 5000 cycles.

1. **T****EM images of Co_3_O_4_@Co-MOF after cycling for 5000 cycles**

**
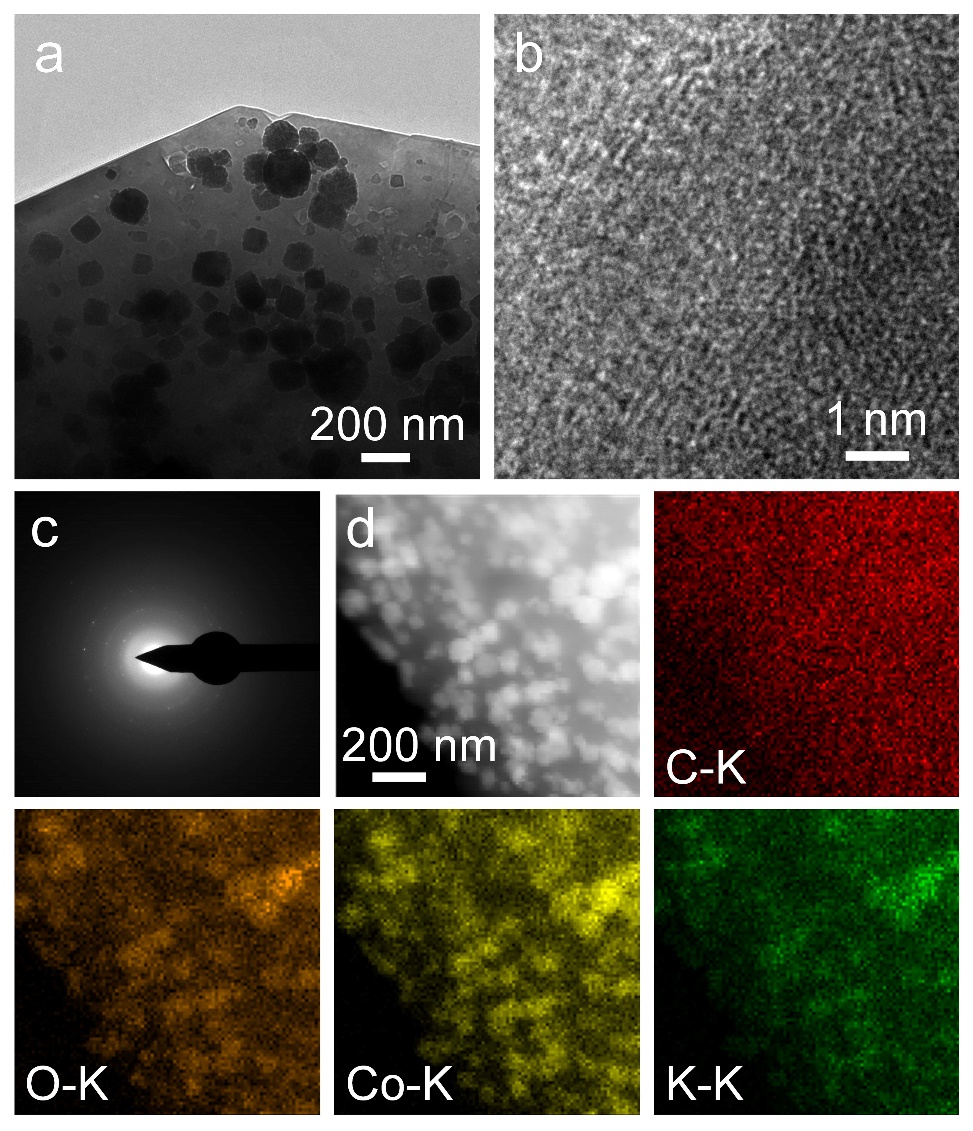
**

**Figure S20.** a) TEM image, b) HRTEM image, c) SAED pattern, and d) STEM image and elemental mapping of C-K, O-K, Co-K, K-K of Co_3_O_4_@Co-MOF after cycling for 5000 cycles.

1. **EDS of Co_3_O_4_@Co-MOF after cycling for 5000 cycles**

**
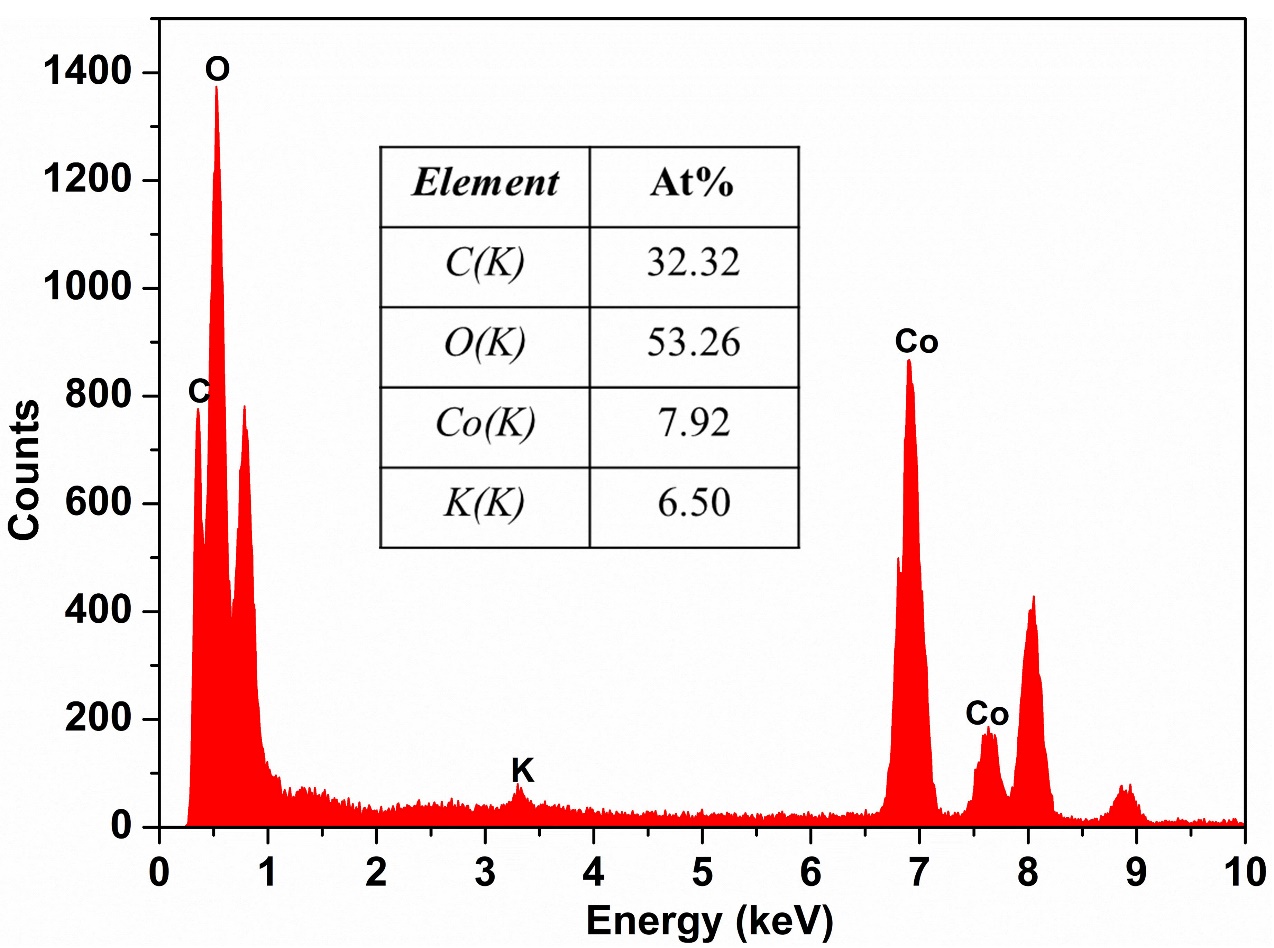
**

**Figure S21.** EDS analysis of Co_3_O_4_@Co-MOF after cycling for 5000 cycles.

1. **Optical and SEM images of Co-MOF after immersion in 3.0 M KOH for 0 h, 24 h, 7 days and 15 days**

**
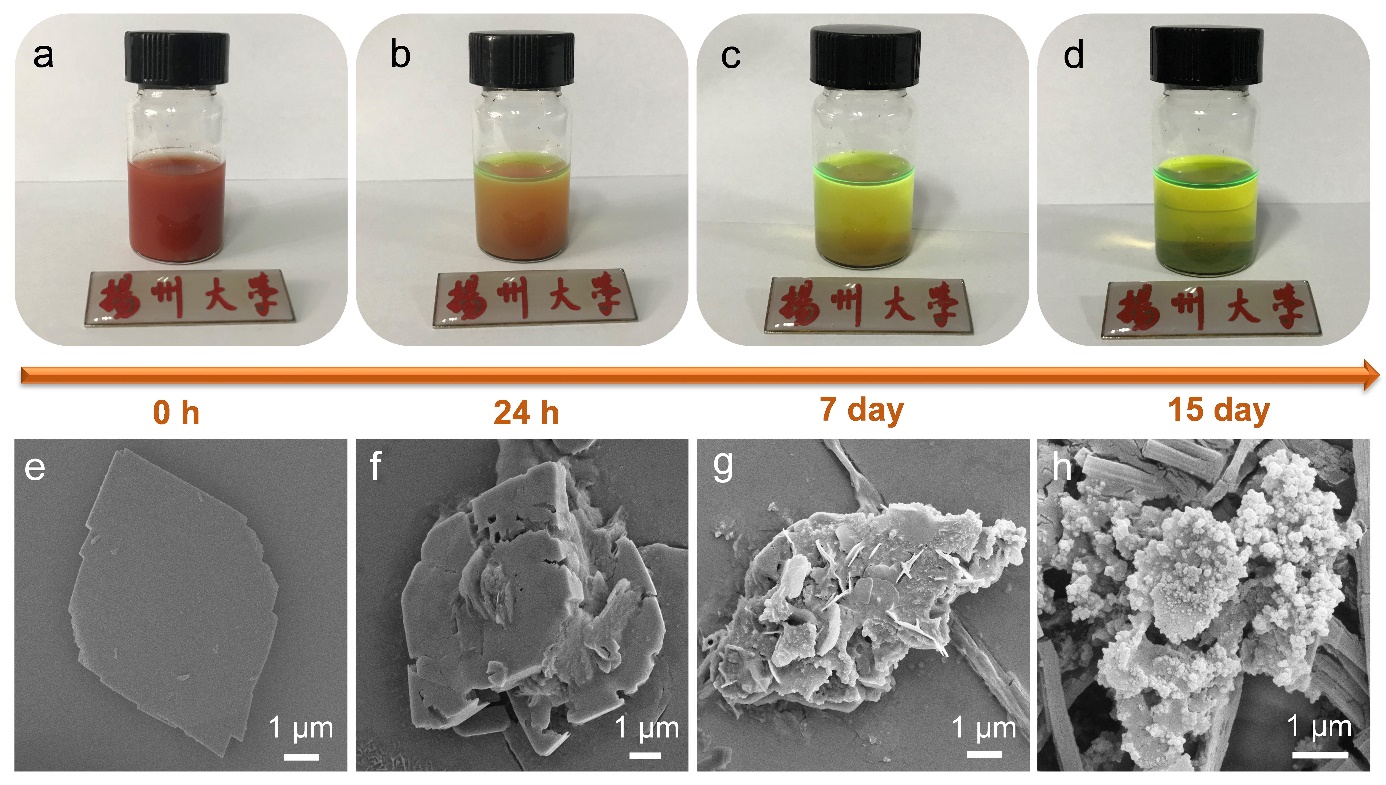
**

**Figure S22.** a-d) Optical images of Co-MOF after immersion in 3.0 M KOH for 0 h, 24 h, 7 days and 15 days, e-h) the corresponding SEM images.

1. **XRD patterns of Co-MOF for 0 h and 15 days.**

**

**

**Figure S23.** XRD patterns of Co-MOF after immersion in 3.0 M KOH for 0 h and 15 days.

1. **SEM images of Co-MOF and Co_3_O_4_@Co-MOF after immersion** **in HNO_3_ solution**


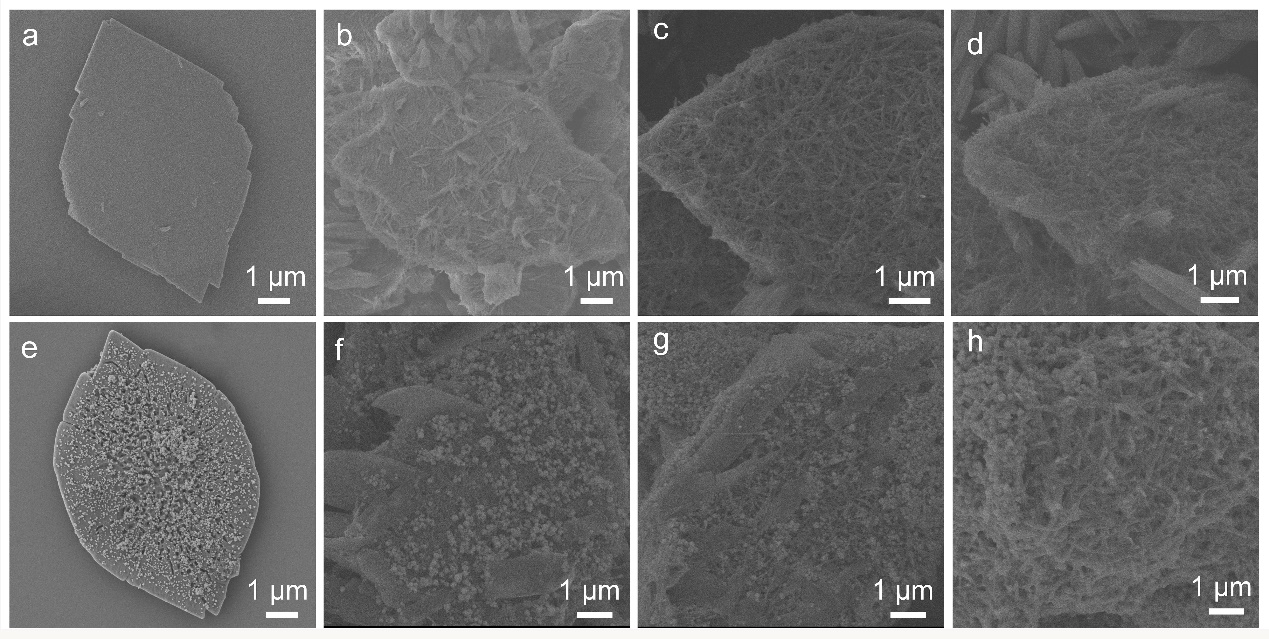


**Figure S24.** a-d) SEM images of Co-MOF after immersion in different concentrations of HNO_3_ solution for 48 h: a) 0% HNO_3_, b) 2.5% HNO_3_, c) 20% HNO_3_, d) 50% HNO_3_. e-h) SEM images of Co_3_O_4_@Co-MOF after immersion in different concentrations of HNO_3_ solution for 48 h: e) 0% HNO_3_, f) 2.5% HNO_3_, g) 20% HNO_3_, h) 50% HNO_3_.

1. **SEM images of Co-MOF and Co_3_O_4_@Co-MOF after immersion in HNO_3_ solution**


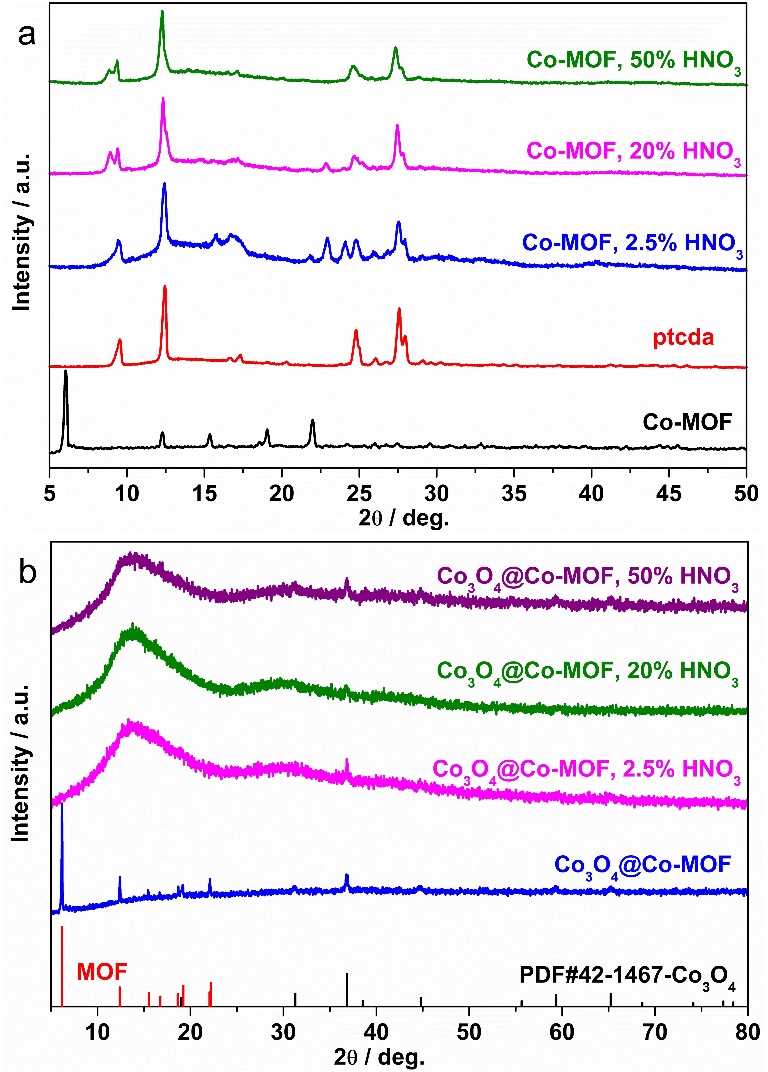


**Figure S25.** a) XRD patterns of Co-MOF after immersion in different concentrations of HNO_3_ solution for 48 h. b) XRD patterns of Co_3_O_4_@Co-MOF after immersion in different concentrations of HNO_3_ solution for 48 h.

1. **XPS spectra of Co_3_O_4_@Co-MOF, Co_3_O_4_@Co-MOF after immersed in 3.0 M KOH for 15 days and after cycling for 5000 cycles**

**
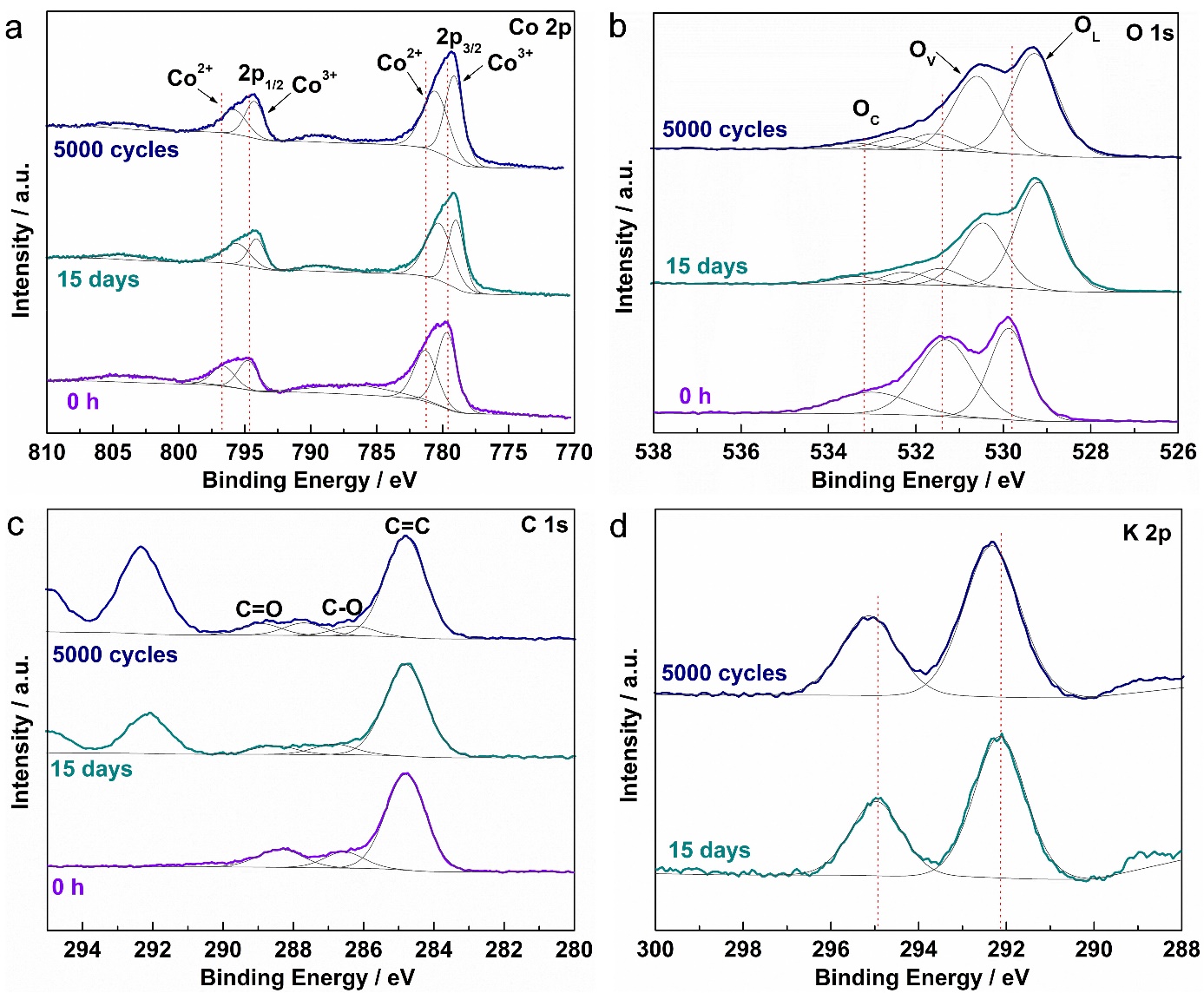
**

**Figure S26.** XPS spectra of Co_3_O_4_@Co-MOF, Co_3_O_4_@Co-MOF after immersed in 3.0 M KOH at room temperature for 15 days and after cycling for 5000 cycles. a) Co 2p, b) O 1s, c) C 1s and d) K 2p.

As shown in **Figure S26**, after cycling test, the Co 2p and O 1s peaks only had a ≈0.6 eV downshift. Although Co 2p and O 1s spectrum only had a ≈0.6 eV downshift, the peaks still correspond to the characteristic of Co_3_O_4_@Co-MOF, indicating that the valences and chemical states of Co and O remained the same.

1. **The GCD curves of activated carbon**

**
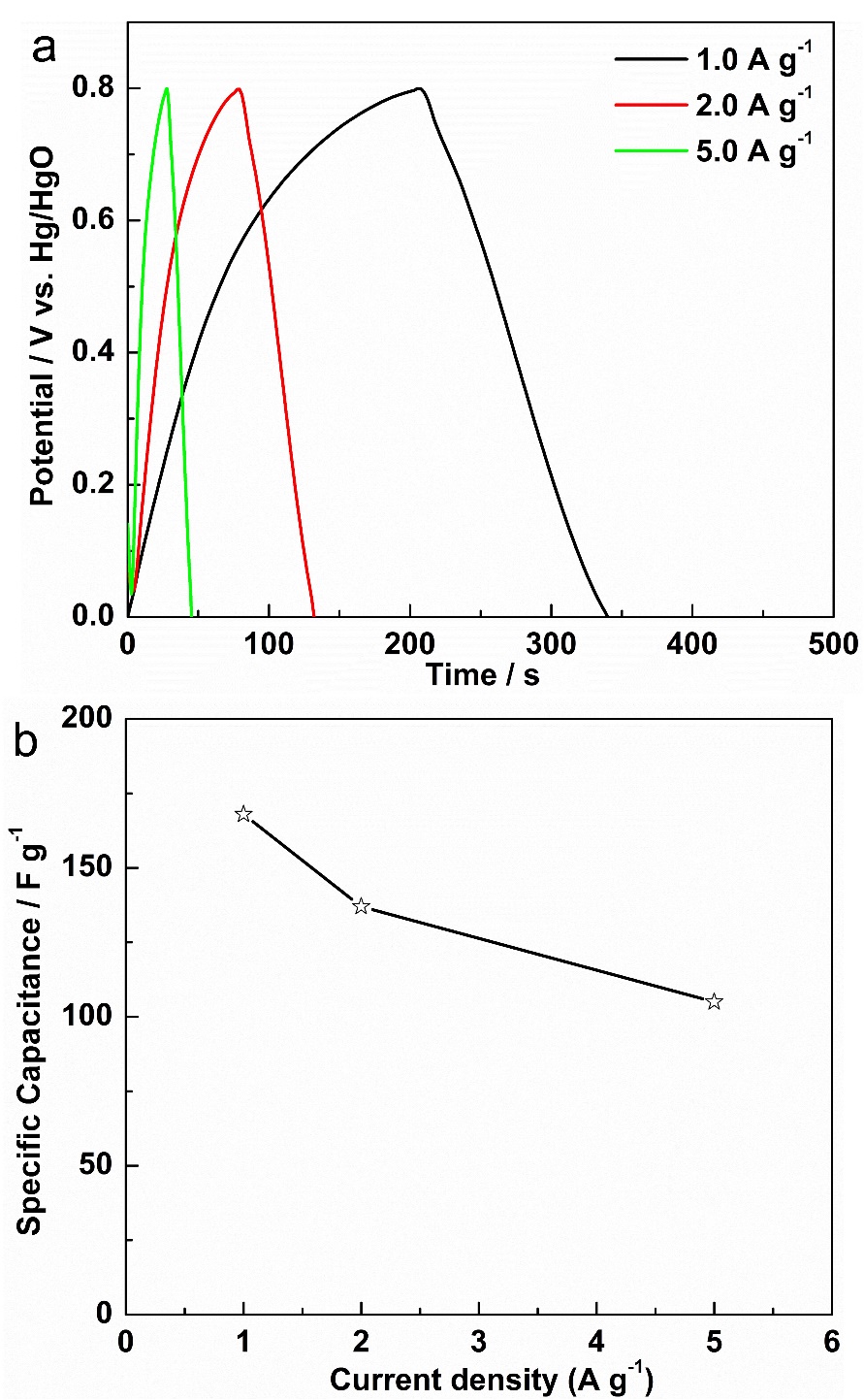
**

**Figure S27.** a) The GCD curves, and b) specific capacitances of the activated carbon.

1. **CV curves of the aqueous device with a scan rate at 50 mV s^-1^ at different potentials**

**
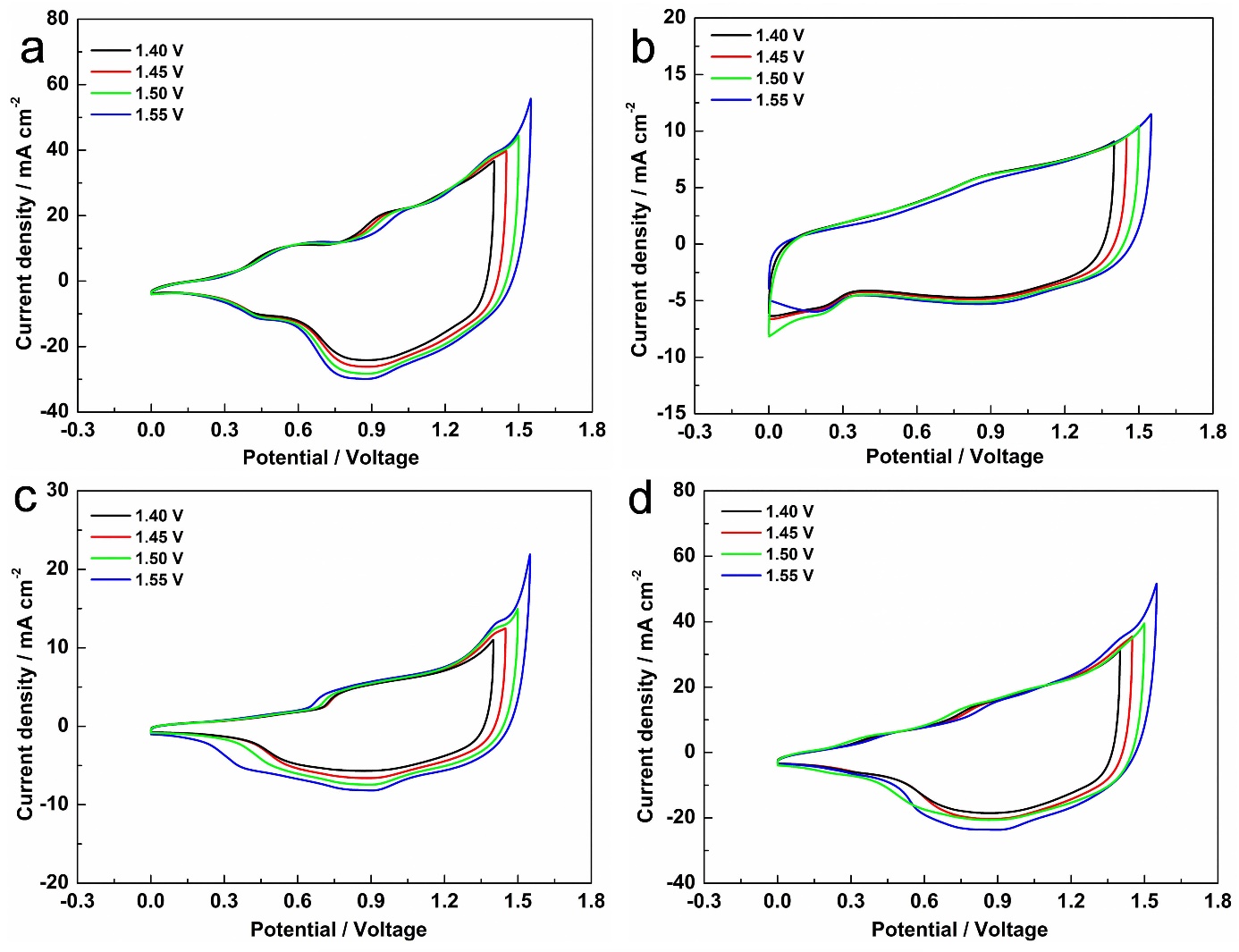
**

**Figure S28.** CV curves of the as-prepared aqueous devices with a scan rate of 50 mV s^-1^ at different potentials: a) Co_3_O_4_@Co-MOF, b) Co-MOF, c) Co_3_O_4_, and d) Co_3_O_4_+Co-MOF.

1. **CV curves of the aqueous device at different scan rates**

**
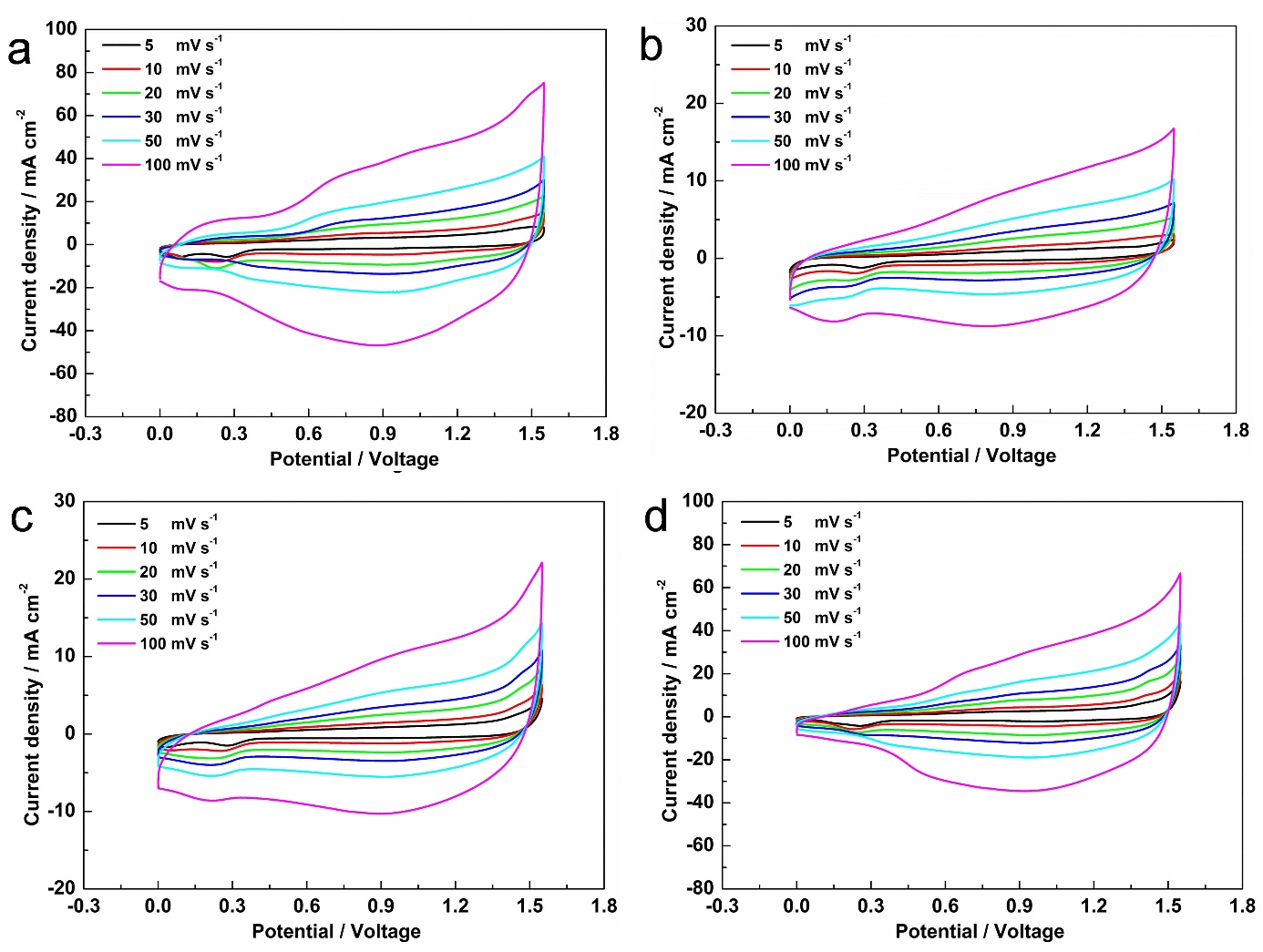
**

**Figure S29.** CV curves of the as-prepared aqueous devices at different scan rates: a) Co_3_O_4_@Co-MOF, b) Co-MOF, c) Co_3_O_4_, and d) Co_3_O_4_+Co-MOF.

1. **The GCD curves of the aqueous device at different current densities**

**
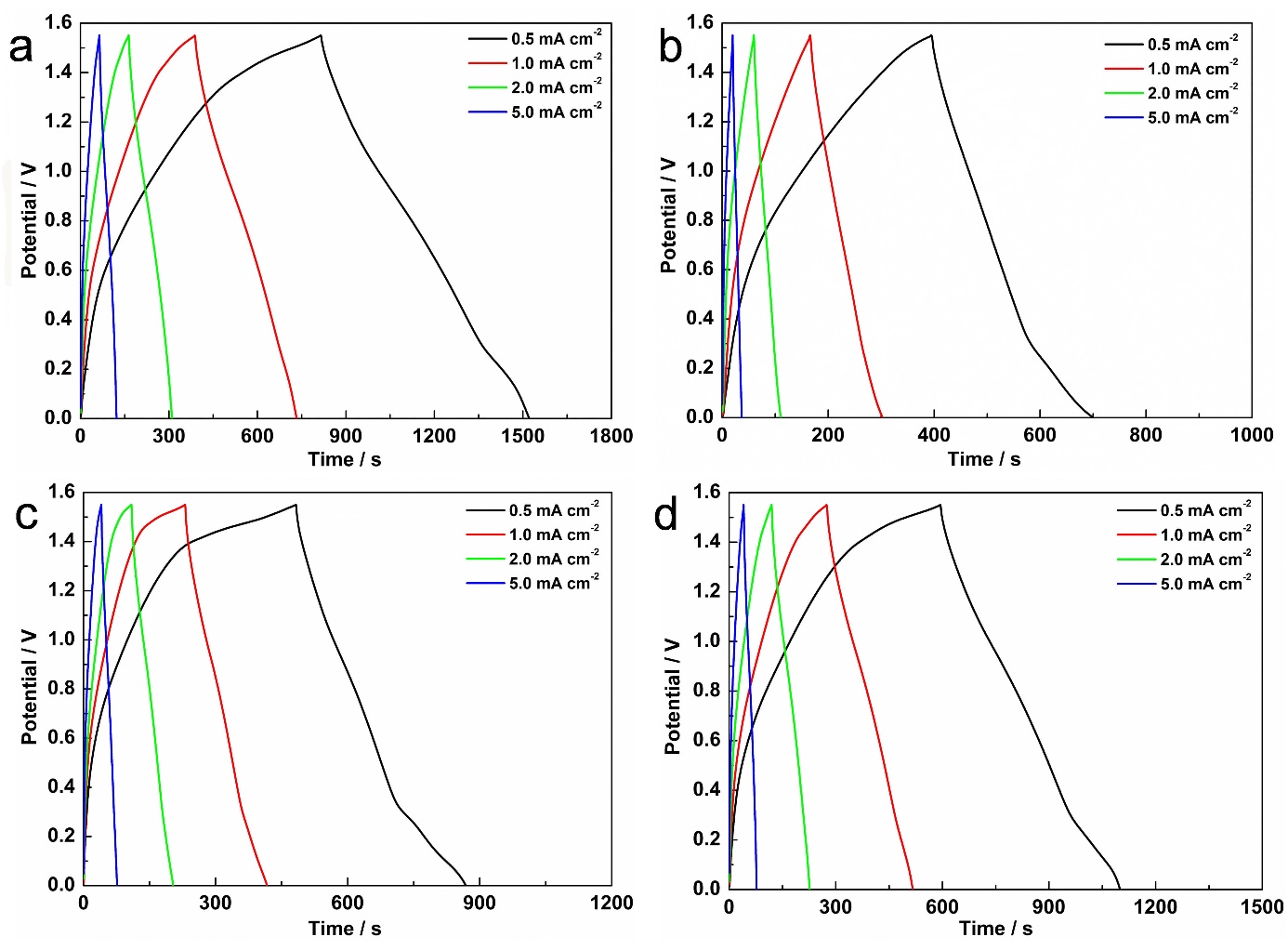
**

**Figure S30.** The GCD curves of the as-prepared aqueous devices at different current densities: a) Co_3_O_4_@Co-MOF, b) Co-MOF, c) Co_3_O_4_, and d) Co_3_O_4_+Co-MOF.

1. **Electrochemical characterization of the aqueous device**

**
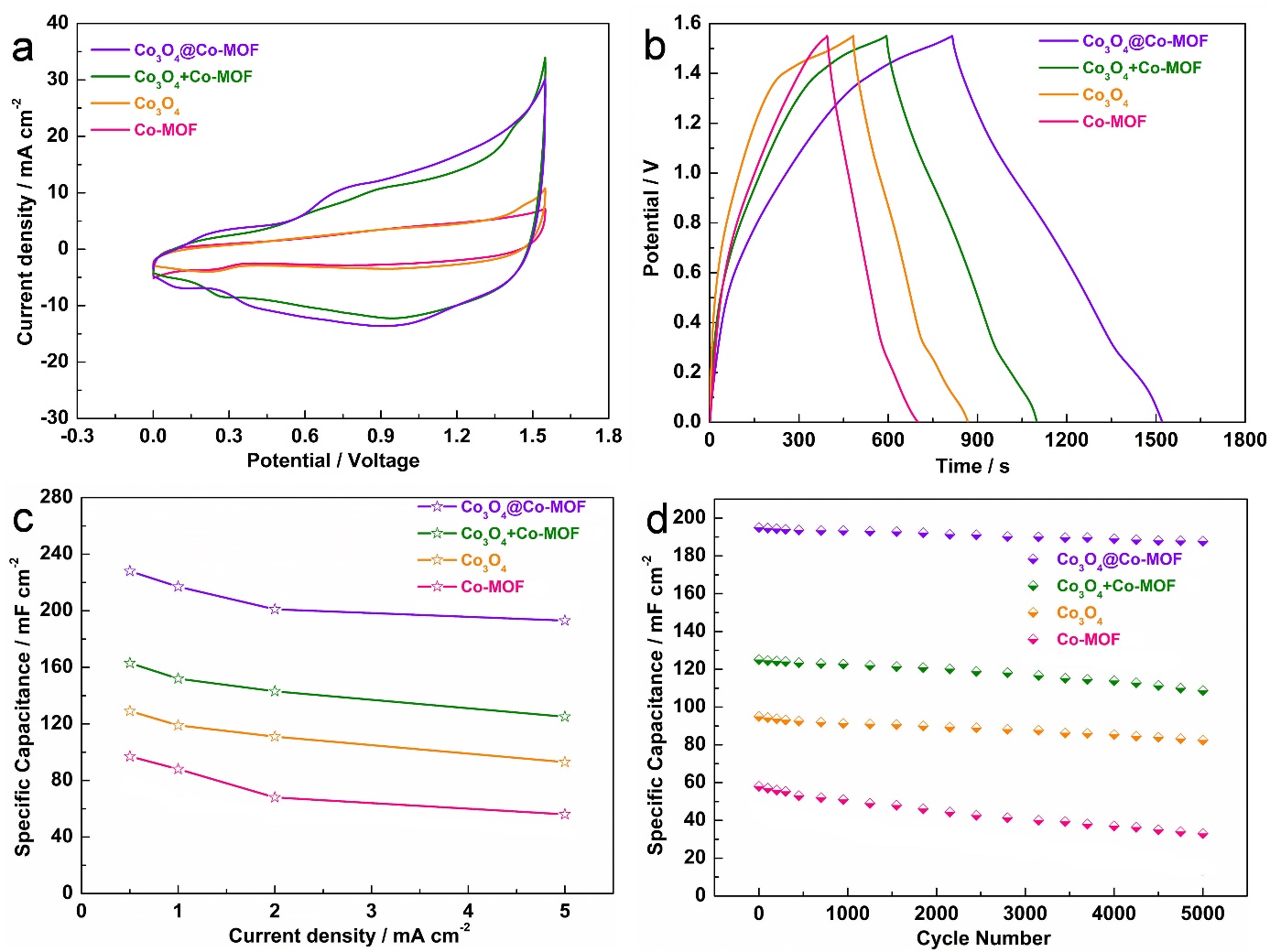
**

**Figure S31.** Electrochemical characterization of the as-prepared aqueous devices: a) CV curves with a scan rate of 30 mV s^-1^. b) GCD curves at a current density of 0.5 mA cm^-2^. c) The specific capacitance changing vs. current density, and d) cycling performance at 5 mA cm^-2^ for 5000 cycles.

1. **The thickness of the electrode**

**
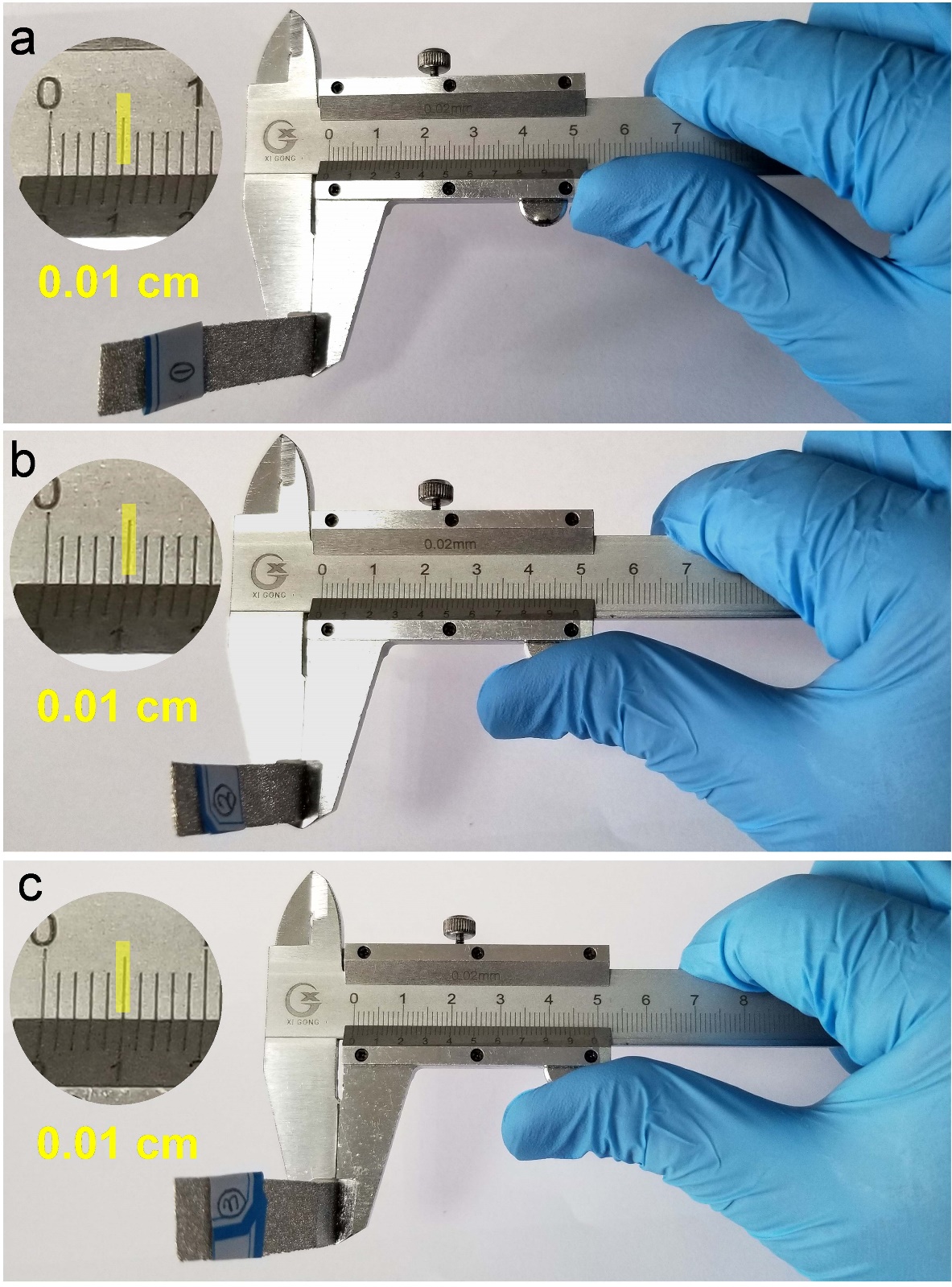
**

**Figure S32.** Thickness measurement of the electrode after pressing. a) Co_3_O_4_@Co-MOF/NF-1, b) Co_3_O_4_@Co-MOF/NF-2, and c) Co_3_O_4_@Co-MOF/NF-3.

1. **The electrochemical impedance spectra of the aqueous device**

**
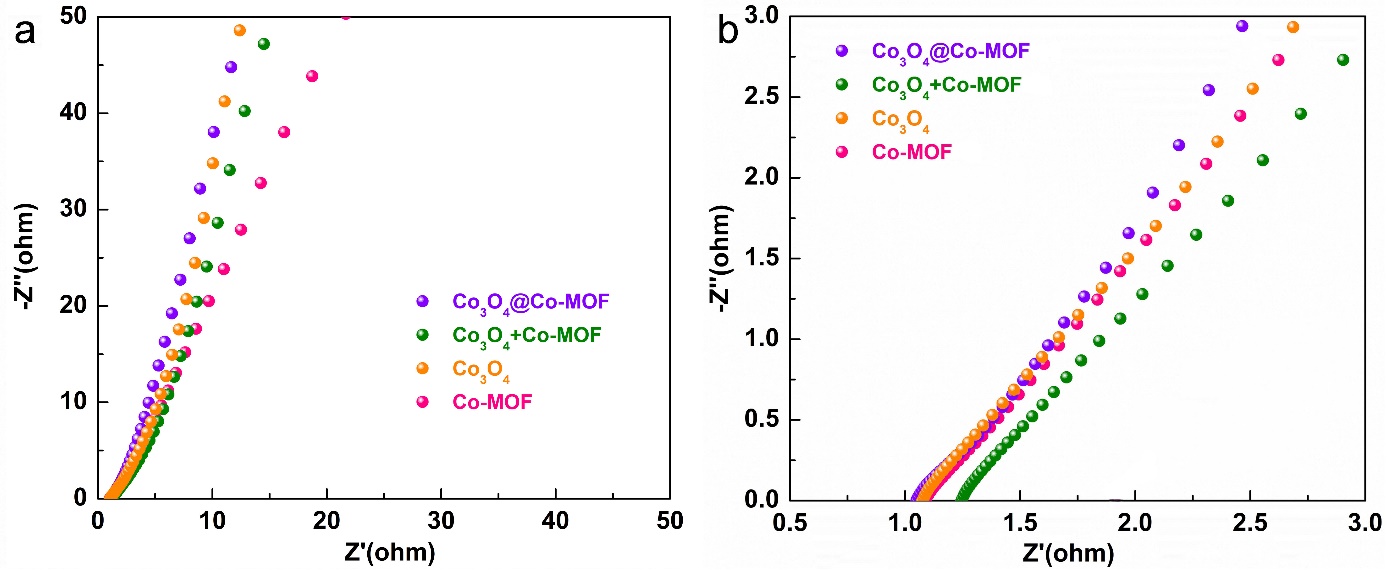
**

**Figure S33.** The electrochemical impedance spectra of the as-prepared aqueous devices at room temperature.

1. **CV curves of the solid-state ﬂexible device at different potentials**

**
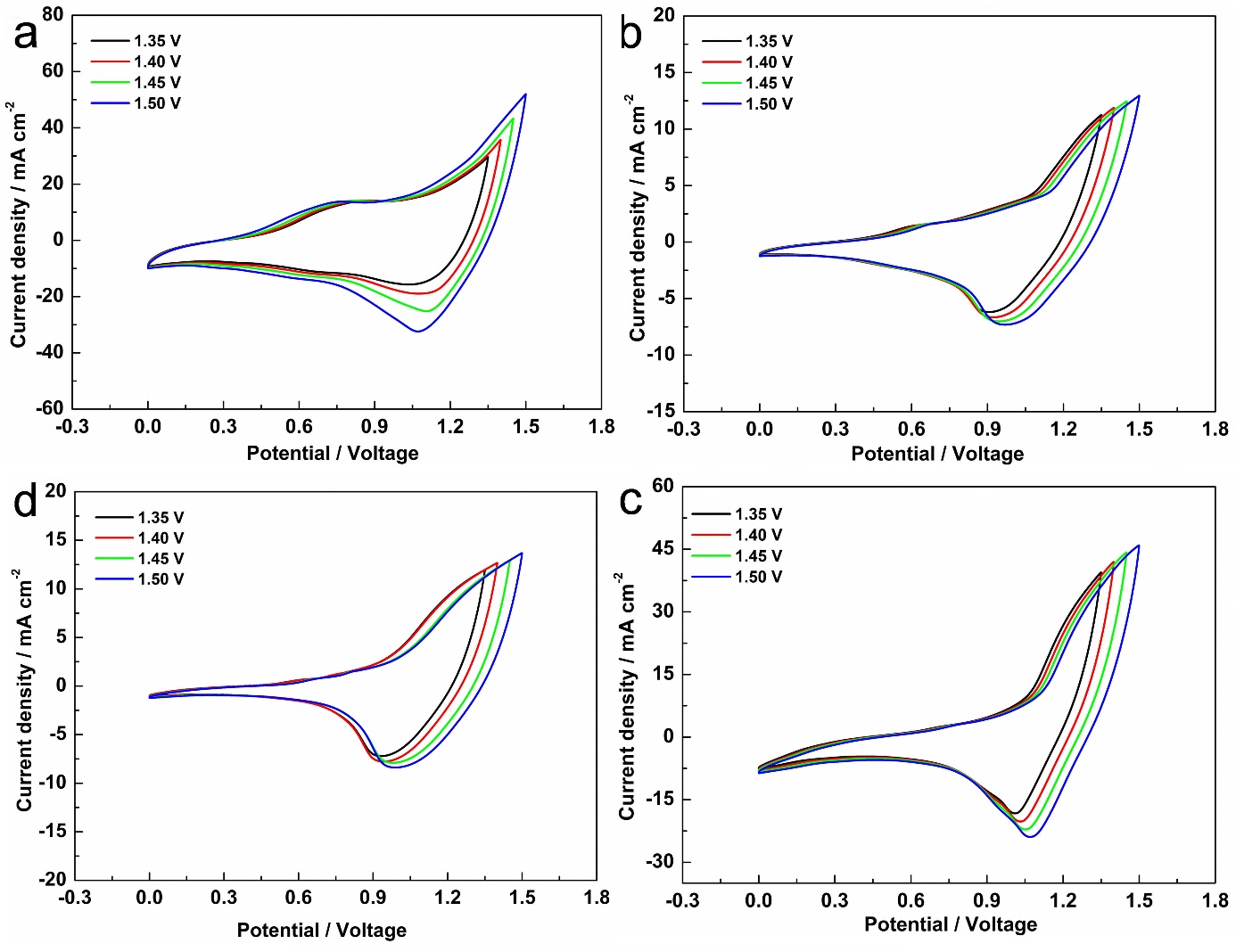
**

**Figure S34.** CV curves of the as-prepared solid-state ﬂexible devices with a scan rate of 50 mV s^-1^ at different potentials: a) Co_3_O_4_@Co-MOF, b) Co-MOF, c) Co_3_O_4_, and d) Co_3_O_4_+Co-MOF.

1. **CV curves of the solid-state ﬂexible device at different scan rates**

**
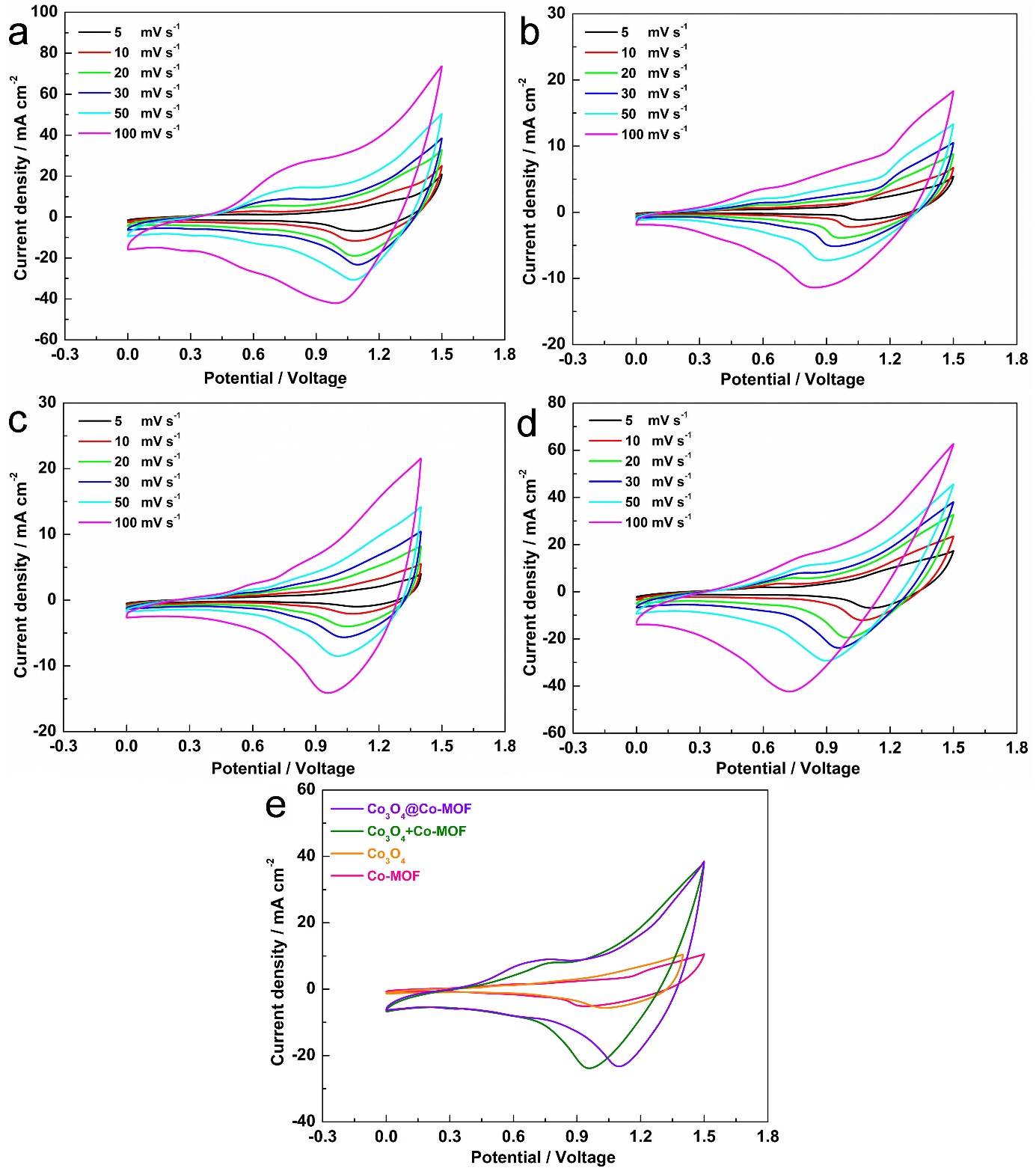
**

**Figure S35.** CV curves of the as-prepared solid-state ﬂexible devices at different scan rates: a) Co_3_O_4_@Co-MOF, b) Co-MOF, c) Co_3_O_4_, d) Co_3_O_4_+Co-MOF, and e) CV curves with a scan rate of 30 mV s^-1^.

1. **The GCD curves of the solid-state ﬂexible device at different potentials**

**
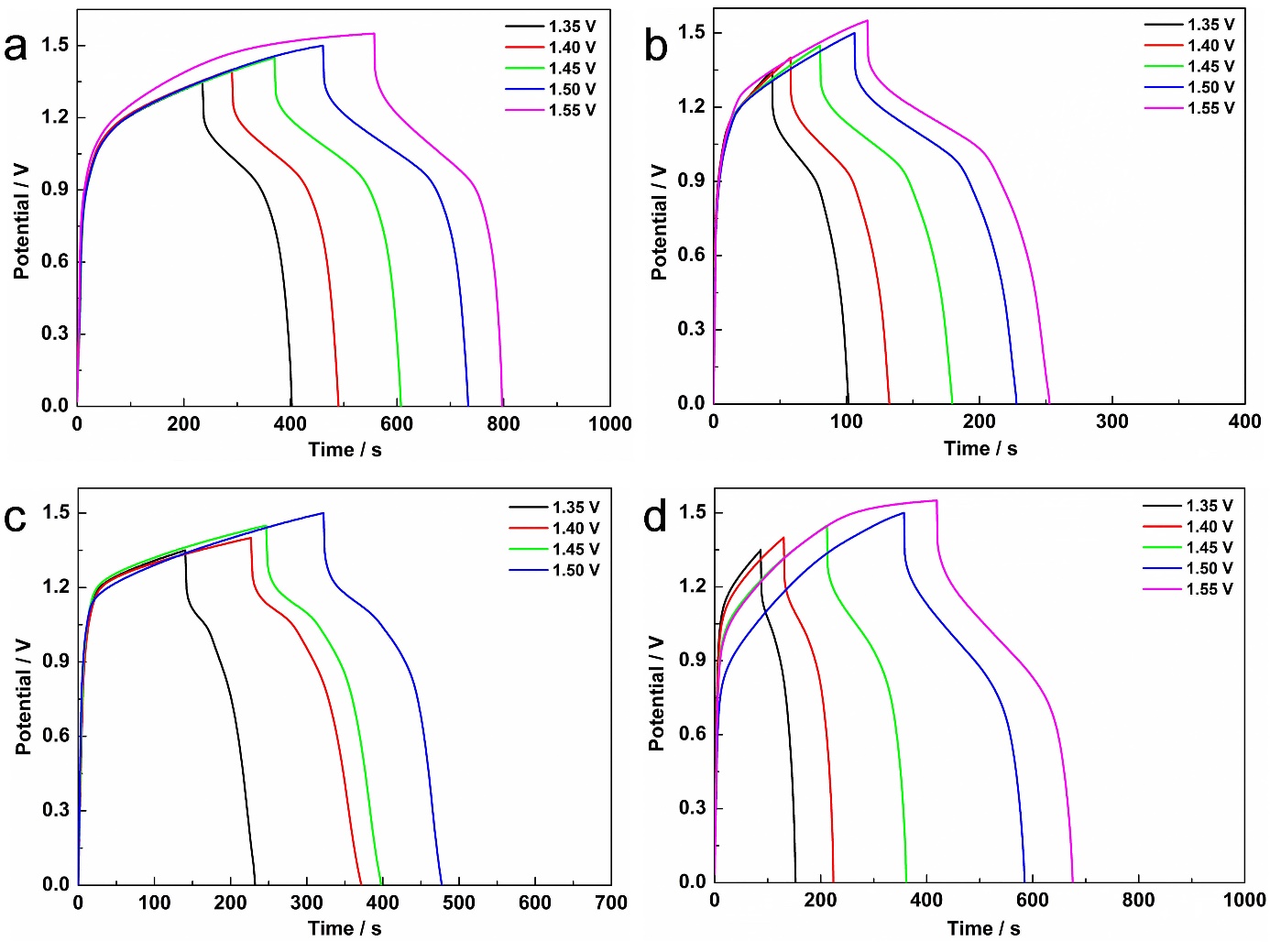
**

**Figure S36.** The GCD curves of the as-prepared solid-state ﬂexible devices with a current density 1 mA cm^-2^ at different potentials: a) Co_3_O_4_@Co-MOF, b) Co-MOF, c) Co_3_O_4_, and d) Co_3_O_4_+Co-MOF.

1. **The GCD curves of the solid-state ﬂexible device at different current densities**

**
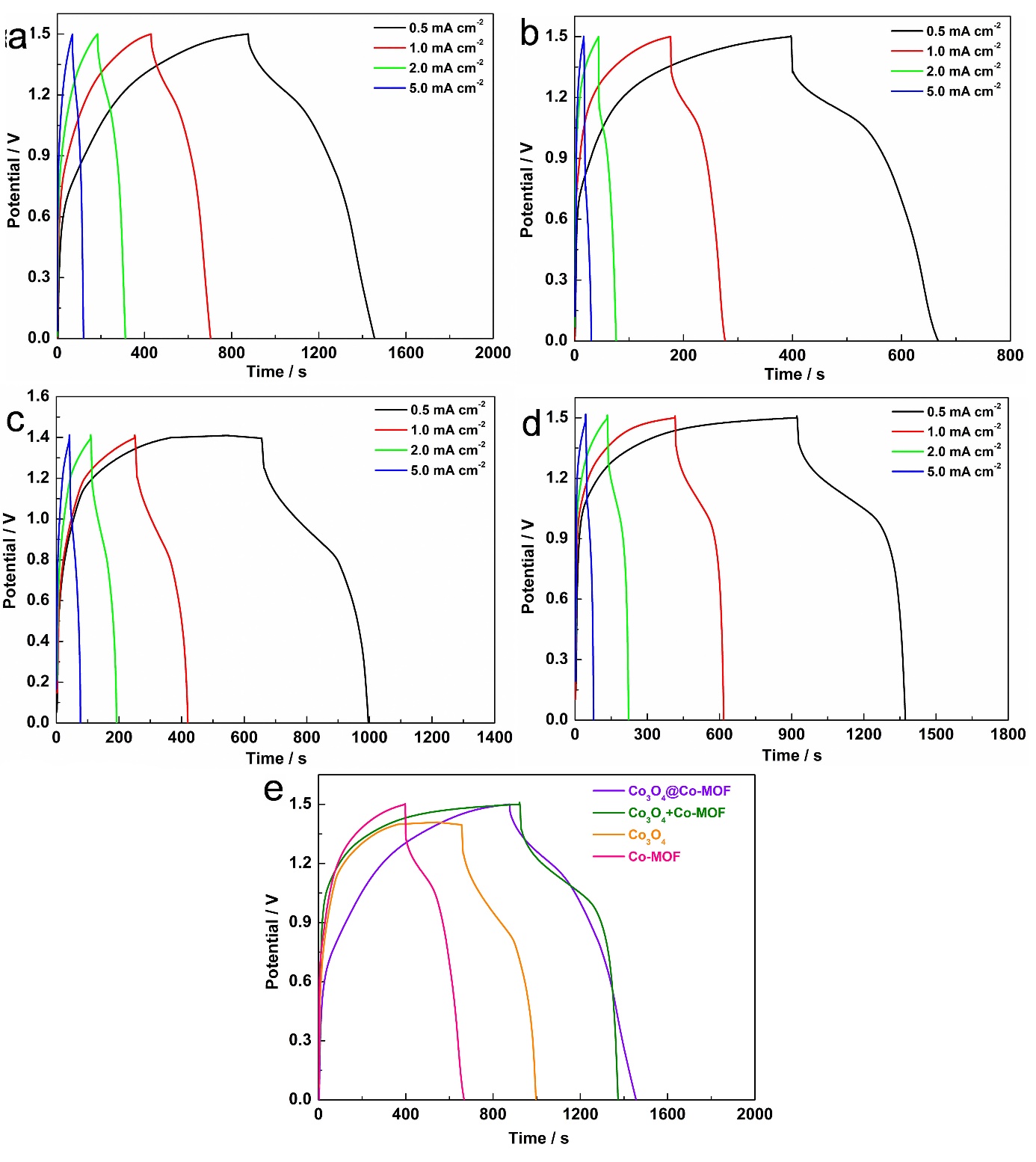
**

**Figure S37.** The GCD curves of the as-prepared solid-state ﬂexible devices at different current densities: a) Co_3_O_4_@Co-MOF, b) Co-MOF, c) Co_3_O_4_, d) Co_3_O_4_+Co-MOF, and e) GCD curves at a current density 0.5 mA cm^-2^.

1. **The electrochemical impedance spectra of the solid-state ﬂexible device**

**
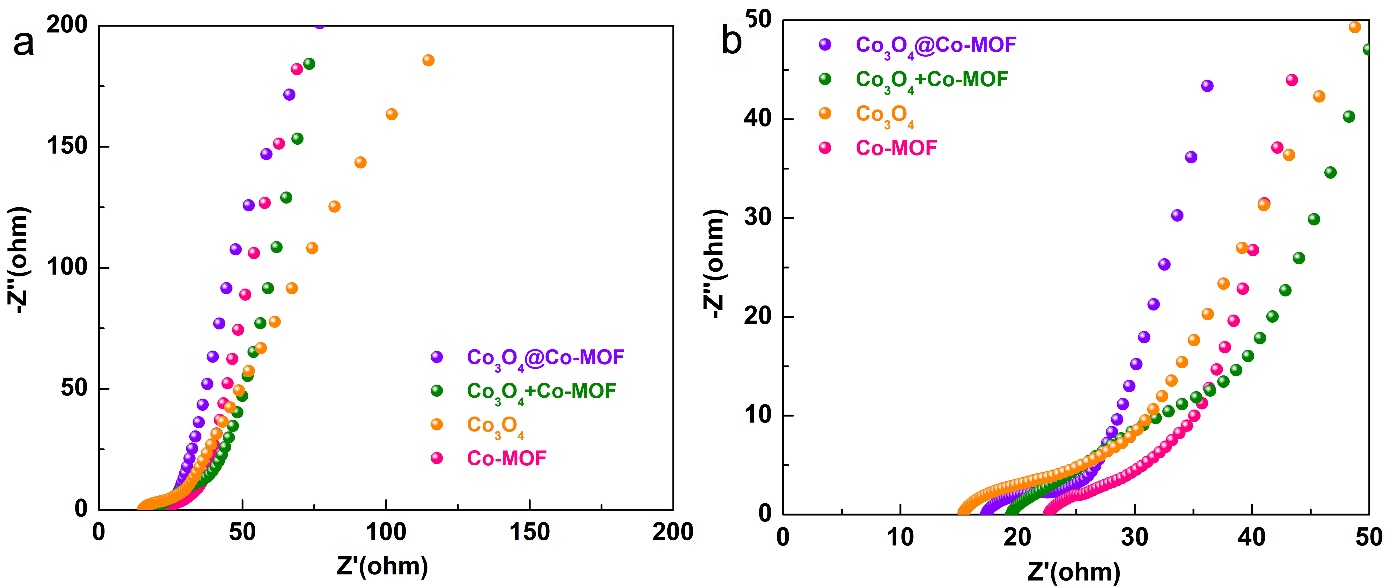
**

**Figure S38.** The electrochemical impedance spectra of the as-prepared solid-state ﬂexible devices at room temperature.

1. **TEM images of Co_3_O_4_@Co-MOF after 400 bending cycles**

**
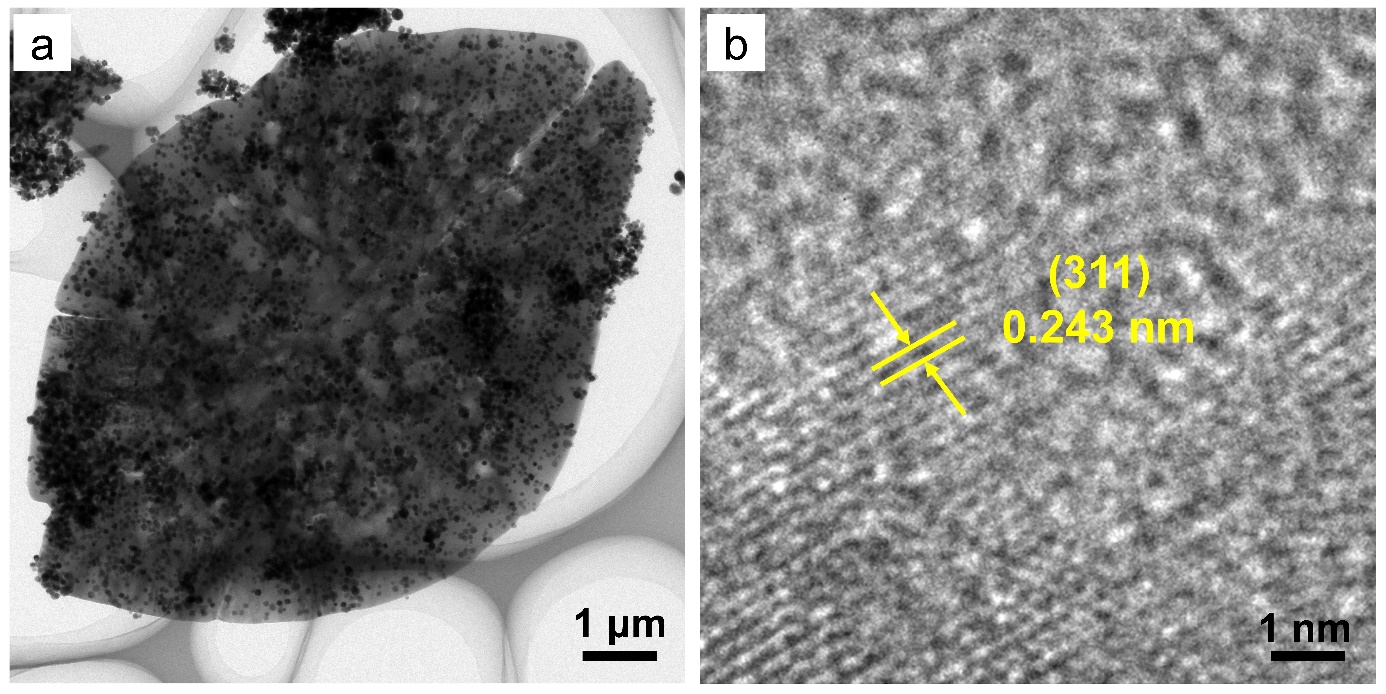
**

**Figure S39.** a) TEM image, and b) HRTEM image of Co_3_O_4_@Co-MOF after 400 bending cycles.

1. **Table S3. Comparison of the obtained materials.**

| **Three-electrode system** | | |
| --- | --- | --- |
| **Material** | **C^a)^ [F g^-1^]**  **(LCD)^b)^** | **C^a)^ [F g^-1^]**  **(HCD)^c)^** |
| Co_3_O_4_ | 479 (0.5 A g^-1^) | 306 (8 A g^-1^)  110 (8 A g^-1^)  408 (8 A g^-1^)  861 (8 A g^-1^) |
| Co-MOF | 356 (0.5 A g^-1^) |  |
| Co_3_O_4_+Co-MOF | 606 (0.5 A g^-1^) |  |
| Co_3_O_4_@Co-MOF | 1020 (0.5 A g^-1^) |  |
| **The aqueous electrochemical energy storage device** | | |
| **Material** | **C^a)^ [mF cm^-2^]**  **area capacitance** | **C^a)^ [F cm^-3^]**  **volume capacitance**  12.9 (0.05 A cm^-3^)  9.7 (0.05 A cm^-3^)  16.3 (0.05 A cm^-3^)  22.8 (0.05 A cm^-3^) |
| Co_3_O_4_ | 129 (0.5 mA cm^-2^) |  |
| Co-MOF | 97 (0.5 mA cm^-2^) |  |
| Co_3_O_4_+Co-MOF | 163 (0.5 mA cm^-2^) |  |
| Co_3_O_4_@Co-MOF | 228 (0.5 mA cm^-2^) |  |
| **The solid-state flexible electrochemical energy storage device** | | |
| **Material** | **C^a)^ [mF cm^-2^]**  **area capacitance** | **C^a)^ [F cm^-3^]**  **volume capacitance**  12.3 (0.05 A cm^-3^)  8.7 (0.05 A cm^-3^)  15.1 (0.05 A cm^-3^)  19.2 (0.05 A cm^-3^) |
| Co_3_O_4_ | 123 (0.5 mA cm^-2^) |  |
| Co-MOF | 87 (0.5 mA cm^-2^) |  |
| Co_3_O_4_+Co-MOF | 151 (0.5 mA cm^-2^) |  |
| Co_3_O_4_@Co-MOF | 192 (0.5 mA cm^-2^) |  |

a) C: capacitance; b) LCD: low current density; c) HCD: high current density.

1. **Table S4. A comparison with previously reported metal oxide nanomaterials.**

Table S4. A comparison with previously reported metal oxide nanomaterials.

| **Material** | **SA^a)^**  **[m^2^ g^-1^]** | **C^b)^ [F g^-1^]**  **(LCD)^c)^** | **C^b)^ [F g^-1^]**  **(HCD)^d)^** | **Capacity retention [%]** | **Electrolyte** | **Ref.** |
| --- | --- | --- | --- | --- | --- | --- |
| Co_3_O_4_ nanoparticles | 50.12 | 189.1 (0.2 A g^-1^) | 148.4 (1 A g^-1^) | — | 1 M KOH | [1] |
| Co_3_O_4_ nanotubes | 36.2 | 647 (1 A g^-1^) | 515 (6 A g^-1^) | — | 2 M KOH | [2] |
| Dendrite-like Co_3_O_4_ | — | 207.8 (0.5 A g^-1^) | 75 (6 A g^-1^) | 97.5 (1000 cycles, 1.8 A g^-1^) | 3 M KOH | [3] |
| Co_3_O_4_ nanowires | — | 599 (2 A g^-1^) | 439 (40 A g^-1^) | 91 (7500 cycles, 2 A g^-1^) | 1 M KOH | [4] |
| Co_3_O_4_ particles | 47.12 | 137.2 (0.5 A g^-1^) | 119 (3 A g^-1^) | — | 2 M KOH | [5] |
| Co_3_O_4_ nanosheets | 53.1 | 786 (1 A g^-1^) | 350 (40 A g^-1^) | 89 (5000 cycles, 10 A g^-1^) | 3 M KOH | [6] |
| Co_3_O_4_ nanoparticles | 21.5 | 208 (1 A g^-1^) | 102 (3 A g^-1^) | 97 (1000 cycles, 1 A g^-1^) | 6 M KOH | [7] |
| Ni_x_Co_3-x_O_4-1_ nanoparticles | 92 | 797 (1 A g^-1^) | 597 (20 A g^-1^) | 75 (10000 cycles, 10 A g^-1^) | 6 M KOH | [8] |
| Double-shelled NiO/ZnO | 36.4 | 497 (1.3 A g^-1^) | 298 (13.3 A g^-1^) | — | 3 M KOH | [9] |
| Co_3_O_4_@MnO_2_ | — | 480 (2.67 A g^-1^) | — | 97.3 (5000 cycles, 2.67 A g^-1^) | 1 M LiOH | [10] |
| Co_3_O_4_@PPy@MnO_2_ | — | 782 (0.5 A g^-1^) | 417 (40 A g^-1^) | 97.6 (2000 cycles, 5 A g^-1^) | 1 M KOH | [11] |
| Co_3_O_4_/graphene | — | 978.1 (1 A g^-1^) | 916.5 (10 A g^-1^) | 99.3 (20000 cycles, 10 A g^-1^) | 2 M KOH | [12] |
| Co_3_O_4_/N-doped carbon | 232 | 581 (1 A g^-1^) | 532 (20 A g^-1^) | — | 2 M KOH | [13] |
| **Co_3_O_4_@Co-MOF** | **453.5** | **1020 (0.5 A g^-1^)** | **861 (8 A g^-1^)** | **96.9 (5000 cycles, 5 A g^-1^)** | **3 M KOH** | **This work** |

a) SA: surface area; b) C: capacitance; c) LCD: low current density; d) HCD: high current density.

1. **Table S5. A comparison with previously reported MOF nanomaterials.**

Table S5. A comparison with previously reported MOF nanomaterials.

| **Material** | **SA^a)^**  **[m^2^ g^-1^]** | **C^b)^ [F g^-1^]**  **(LCD/LSR)^c)^** | **C^b)^ [F g^-1^]**  **(HCD/HSR)^d)^** | **Capacity retention**  **[%]** | **Electrolyte** | **Ref.** |
| --- | --- | --- | --- | --- | --- | --- |
| Co8-MOF-5 | 2900 | 0.49 (25 mV s^-1^) | 0.3 (10 mA g^-1^) | 92 (1000 cycles,  10 mA g^-1^) | 0.1 M TBAPF_6_ | [14] |
| Co-BPDC | 138.35 | 179.2 (10 mV s^-1^) | 60 (200 mV s^-1^) | 77.4 (1000 cycles, 100 mV s^-1^) | 0.5 M LiOH | [15] |
| Co-MOF-71 | — | 206.76 (0.6 A g^-1^) | — | 98.5 (1000 cycles, —) | 1 M LiOH | [16] |
| Ni-MOF | 148 | 634 (5 mV s^-1^) | 457 (10 mV s^-1^) | 84 (2000 cycles,  50 mV s^-1^) | 6 M KOH | [17] |
| Ni_3_(btc)_2_·12H_2_O | — | 726 (1 A g^-1^) | 313.8 (5 A g^-1^) | 65 (5000 cycles,  1 A g^-1^) | 2 M KOH | [18] |
| Ni-DMOF-ADC | 783 | 552 (1 A g^-1^) | 438 (20 A g^-1^) | 98 (16000 cycles, 10 A g^-1^) | 2 M KOH | [19] |
| Ni_3_(HITP)_2_ | 630 | 111 (0.05 A g^-1^) | — | 90 (10000 cycles,  2 A g^-1^) | 1 M TEABF_4_/ACN | [20] |
| Ni-MOF | 117.42 | 998 (1.4 A g^-1^) | 823 (7 A g^-1^) | 96.5 (5000 cycles, 1.4 A g^-1^) | 3 M KOH | [21] |
| MIL-100 (Fe) | — | 39 (5 mV s^-1^) | — | — | 0.1 M K_2_SO_4_ | [22] |
| **Co_3_O_4_@Co-MOF** | **453.5** | **1020 (0.5 A g^-1^)** | **861 (8 A g^-1^)** | **96.9 (5000cycles,**  **5 A g^-1^)** | **3 M KOH** | **This work** |

a) SA: surface area; b) C: capacitance; c) LCD/LSR: low current density/scan rate; d) HCD/HSR: high current density/scan rate.

Co8-MOF-5: a mixed Co-Zn metalbased composition;

TBAPF_6_: tetrabutylammonium hexafluorophosphate;

Co-BPDC: Co- 4,4‘-biphenyldicarboxylate;

Ni-DMOF-ADC: [Ni(9,10-anthracenedicarboxylic acid)(DABCO)_0.5_]

Ni_3_(HITP)_2_: Ni_3_(2,3,6,7,10,11-hexaiminotriphenylene)_2_

1. **Table S6. A comparison with previously reported MOF composites.**

Table S6. A comparison with previously reported MOF composites.

| **Material** | **SA^a)^**  **[m^2^ g^-1^]** | **C^b)^ [F g^-1^]**  **(LCD/LSR)^c)^** | **C^b)^ [F g^-1^]**  **(HCD/HSR)^d)^** | **Capacity retention**  **[%]** | **Electrolyte** | **Ref.** |
| --- | --- | --- | --- | --- | --- | --- |
| Ni_2_CO_3_(OH)_2_/ZIF-8 | 313.8 | 851 (5 mV s^-1^) | 450 (30 mV s^-1^) | — | 6 M KOH | [23] |
| Ni_3_(NO_3_)_2_(OH)_4_@Zr-MOF | 131.2 | 992 (5 mV s^-1^) | 398 (30 mV s^-1^) | 62 (3000 cycles,  10 mV s^-1^) | 6 M KOH | [24] |
| NiC_2_O_4_/ZIF-67 | 261.1 | 1019.7 (5 mV s^-1^) | 573.8 (30 mV s^-1^) | 73 (2000 cycles,  10 mV s^-1^) | 6 M KOH | [25] |
| ZIF-8/GO | 876 | 160 (5 mV s^-1^) | 56 (50 mV s^-1^) | — | 6 M KOH | [26] |
| ZIF-67/GO | 575 | 202 (1 A g^-1^) | — | — | 6 M KOH | [26] |
| rGO-HKUST-1 | 1241 | — | 377 (100 mV s^-1^) | — | 0.5 M Na_2_SO_4_ | [27] |
| CNTs@Mn-MOF | — | 206 (5 mV s^-1^) | 112.5 (100 mV s^-1^) | 88 (3000 cycles,  5 A g^-1^) | 6 M KOH | [28] |
| **Co_3_O_4_@Co-MOF** | **453.5** | **1020 (0.5 A g^-1^)** | **861 (8 A g^-1^)** | **96.9 (5000 cycles, 5 A g^-1^)** | **3 M KOH** | **This work** |

a) SA: surface area; b) C: capacitance; c) LCD/LSR: low current density/scan rate; d) HCD/HSR: high current density/scan rate.

1. **Table S7.** **Comparison of the capacitance value of Co_3_O_4_@Co-MOF at high current densities with previously reported MOF-based materials.**

Table S7. Comparison of the capacitance value of Co_3_O_4_@Co-MOF at high current densities with previously reported MOF-based materials.

| **Material** | **C_m_^a)^ [F g^-1^]** | **Capacity retention at HCD^b)^** | **Ref.** |
| --- | --- | --- | --- |
| Zn-doped Ni-MOF | 1620 | 52% (10 A g^-1^) | [29] |
| Pillared Ni-MOF | 552 | 79% (20 A g-1) | [19] |
| Co-LMOF | 2474 | 41% (20 A g-1) | [30] |
| Ni-MOFs@GO | 2192 | 47% (20 A g-1) | [31] |
| CoNi-MOF | 1104 | 52% (32 A g-1) | [32] |
| **Co_3_O_4_@Co-MOF** | **1020** | **46% (32 A g^-1^)** | **This work** |

a) C_m_: the maximum specific capacitance; b) HCD: high current density.

1. **References**

1 Jiang J, Wei F and Yu G *et al.* Co_3_O_4_ Electrode Prepared by Using Metal-Organic Framework as a Host for Supercapacitors. *J Nanomater* 2015; **2015**:1–6.

2 Li H, Yue F and Yang C *et al.* Porous nanotubes derived from a metal-organic framework as high-performance supercapacitor electrodes. *Ceram Int* 2016; **42**:3121–3129.

3 Pang H, Gao F and Chen Q *et al.* Dendrite-like Co_3_O_4_ nanostructure and its applications in sensors, supercapacitors and catalysis. *Dalt Trans* 2012; **41**:5862.

4 Xia X, Tu J and Mai Y *et al.* Self-supported hydrothermal synthesized hollow Co_3_O_4_ nanowire arrays with high supercapacitor capacitance. *J Mater Chem* 2011; **21**:9319.

5 Meng F, Fang Z and Li Z *et al.* Porous Co_3_O_4_ materials prepared by solid-state thermolysis of a novel Co-MOF crystal and their superior energy storage performances for supercapacitors. *J Mater Chem A* 2013; **1**:7235.

6 Rakhi RB, Chen W and Hedhili MN *et al.* Enhanced Rate Performance of Mesoporous Co_3_O_4_ Nanosheet Supercapacitor Electrodes by Hydrous RuO_2_ Nanoparticle Decoration. *ACS Appl Mater Interfaces* 2014; **6**:4196–4206.

7 Zhang F, Hao L and Zhang L *et al.* Solid-state thermolysis preparation of Co_3_O_4_ nano/micro superstructures from metal-organic framework for supercapacitors. *Int J Electrochem Sci* 2011; **6**:2943–2954.

8 Chen S, Xue M and Li Y *et al.* Rational design and synthesis of Ni_x_Co_3-x_O_4_ nanoparticles derived from multivariate MOF-74 for supercapacitors. *J Mater Chem A* 2015; **3**:20145–20152.

9 Li GC, Liu PF and Liu R *et al.* MOF-derived hierarchical double-shelled NiO/ZnO hollow spheres for high-performance supercapacitors. *Dalt Trans* 2016; **45**:13311–13316.

10 Liu J, Jiang J and Cheng C *et al.* Co_3_O_4_ Nanowire@MnO_2_ Ultrathin Nanosheet Core/Shell Arrays: A New Class of High-Performance Pseudocapacitive Materials. *Adv Mater* 2011; **23**:2076–2081.

11 Wang B, He X and Li H *et al.* Optimizing the charge transfer process by designing Co_3_O_4_@PPy@MnO_2_ ternary core-shell composite. *J Mater Chem A* 2014; **2**:12968–12973.

12 Yang S, Liu Y and Hao Y *et al.* Oxygen-Vacancy Abundant Ultrafine Co_3_O_4_/Graphene Composites for High-Rate Supercapacitor Electrodes. *Adv Sci* 2018; **5**:1700659.

13 Liu T, Zhang L and You W *et al.* Core-Shell Nitrogen-Doped Carbon Hollow Spheres/Co_3_O_4_ Nanosheets as Advanced Electrode for High-Performance Supercapacitor. *Small* 2018; **14**:1702407.

14 Díaz R, Orcajo MG and Botas JA *et al.* Co8-MOF-5 as electrode for supercapacitors. *Mater Lett* 2012; **68**:126–128.

15 Lee DY, Shinde DV and Kim EK *et al.* Supercapacitive property of metal-organic-frameworks with different pore dimensions and morphology. *Microporous Mesoporous Mater* 2013; **171**:53–57.

16 Lee DY, Yoon SJ and Shrestha NK *et al.* Unusual energy storage and charge retention in Co-based metal-organic-frameworks. *Microporous Mesoporous Mater* 2012; **153**:163–165.

17 Liao C, Zuo Y and Zhang W *et al.* Electrochemical performance of metal-organic framework synthesized by a solvothermal method for supercapacitors. *Russ J Electrochem* 2013; **49**:983–986.

18 Kang L, Sun SX and Kong LB *et al.* Investigating metal-organic framework as a new pseudo-capacitive material for supercapacitors. *Chinese Chem Lett* 2014; **25**:957–961.

19 Qu C, Jiao Y, and Zhao B *et al.* Nickel-based pillared MOFs for high-performance supercapacitors: Design, synthesis and stability study. *Nano Energy* 2016; **26**:66–73.

20 Sheberla D, Bachman JC and Elias JS *et al.* Conductive MOF electrodes for stable supercapacitors with high areal capacitance. *Nat Mater* 2017; **16**:220–224.

21 Yan Y, Gu P and Zheng S *et al.* Facile synthesis of an accordion-like Ni-MOF superstructure for high-performance flexible supercapacitors. *J Mater Chem A* 2016; **4**:19078–19085.

22 Wang R, Yan X and Lang J *et al.* A hybrid supercapacitor based on flower-like Co(OH)_2_ and urchin-like VN electrode materials. *J Mater Chem A* 2014; **2**:12724.

23 Gao Y, Wu J and Zhang W *et al.* Synthesis of nickel carbonate hydroxide/zeolitic imidazolate framework-8 as a supercapacitors electrode. *RSC Adv* 2014; **4**:36366.

24 Li Z, Tan Y and Zhang W *et al.* Flower-like Ni_3_(NO_3_)_2_(OH)_4_@Zr-metal organic framework (UiO-66) composites as electrode materials for high performance pseudocapacitors. *Ionics* 2016; **22**:2545–2551.

25 Gao Y, Wu J and Zhang W *et al.* Synthesis of nickel oxalate/zeolitic imidazolate framework-67 (NiC_2_O_4_/ZIF-67) as a supercapacitor electrode. *New J Chem* 2015; **39**:94–97.

26 Zhang W, Tan Y and Gao Y *et al.* Nanocomposites of zeolitic imidazolate frameworks on graphene oxide for pseudocapacitor applications. *J Appl Electrochem* 2016; **46**:441–450.

27 Srimuk P, Luanwuthi S and Krittayavathananon A *et al.* Solid-type supercapacitor of reduced graphene oxide-metal organic framework composite coated on carbon fiber paper. *Electrochim Acta* 2015; **157**:69–77.

28 Zhang Y, Lin B and Sun Y *et al.* Carbon nanotubes@metal-organic frameworks as Mn-based symmetrical supercapacitor electrodes for enhanced charge storage. *RSC Adv* 2015; **5**:58100–58106.

29 Yang J, Zheng C, and Xiong P *et al.* Zn-doped Ni-MOF material with a high supercapacitive performance. *J Mater Chem A* 2014; **2**:19005–19010.

30 Liu X, Shi C, and Zhai C *et al*. Cobalt-Based Layered Metal-Organic Framework as an Ultrahigh Capacity Supercapacitor Electrode Material. *ACS Appl Mater Interfaces* 2016; **8**:4585–4591.

31 Zhou Y, Mao Z, and Wang W *et al*. In-Situ Fabrication of Graphene Oxide Hybrid Ni-Based Metal-Organic Framework (Ni-MOFs@GO) with Ultrahigh Capacitance as Electrochemical Pseudocapacitor Materials. *ACS Appl Mater Interfaces* 2016; **8**:28904–28916.

32 Deng T, Lu Y, and Zhang W *et al.* Inverted Design for High-Performance Supercapacitor Via Co(OH)_2_ -Derived Highly Oriented MOF Electrodes. *Adv Energy Mater* 2018; **8**:1702294.
